# Ivermectin reverses the drug resistance in cancer cells through EGFR/ERK/Akt/NF-κB pathway

**DOI:** 10.1186/s13046-019-1251-7

**Published:** 2019-06-18

**Authors:** Lu Jiang, Pan Wang, Ying-Jian Sun, Yi-Jun Wu

**Affiliations:** 1 Laboratory of Molecular Toxicology, State Key Laboratory of Integrated Management of Pest Insects and Rodents, Institute of Zoology, Chinese Academy of Sciences, 1-5 Beichenxilu Rd., Chaoyang, Beijing, 100101 China; 2 Department of Veterinary Medicine, Beijing University of Agriculture, Beinonglu Rd, Changping, Beijing, 102206 China; 3 University of Chinese Academy of Sciences, Beijing, 100049 People’s Republic of China

**Keywords:** Drug resistance, Reversal, Ivermectin, P-glycoprotein, EGFR

## Abstract

**Background:**

Discovery and development of novel drugs that are capable of overcoming drug resistance in tumor cells are urgently needed clinically. In this study, we sought to explore whether ivermectin (IVM), a macrolide antiparasitic agent, could overcome the resistance of cancer cells to the therapeutic drugs.

**Methods:**

We used two solid tumor cell lines (HCT-8 colorectal cancer cells and MCF-7 breast cancer cells) and one hematologic tumor cell line (K562 chronic myeloid leukemia cells), which are resistant to the chemotherapeutic drugs vincristine and adriamycin respectively, and two xenograft mice models, including the solid tumor model in nude mice with the resistant HCT-8 cells and the leukemia model in NOD/SCID mice with the resistant K562 cells to investigate the reversal effect of IVM on the resistance *in vitro* and *in vivo*. MTT assay was used to investigate the effect of IVM on cancer cells growth *in vitro*. Flow cytometry, immunohistochemistry, and immunofluorescence were performed to investigate the reversal effect of IVM *in vivo*. Western blotting, qPCR, luciferase reporter assay and ChIP assay were used to detect the molecular mechanism of the reversal effect. Octet RED96 system and Co-IP were used to determine the interactions between IVM and EGFR.

**Results:**

Our results indicated that ivermectin at its very low dose, which did not induce obvious cytotoxicity, drastically reversed the resistance of the tumor cells to the chemotherapeutic drugs both *in vitro* and *in vivo*. Mechanistically, ivermectin reversed the resistance mainly by reducing the expression of P-glycoprotein (P-gp) via inhibiting the epidermal growth factor receptor (EGFR), not by directly inhibiting P-gp activity. Ivermectin bound with the extracellular domain of EGFR, which inhibited the activation of EGFR and its downstream signaling cascade ERK/Akt/NF-κB. The inhibition of the transcriptional factor NF-κB led to the reduced P-gp transcription.

**Conclusions:**

These findings demonstrated that ivermectin significantly enhanced the anti-cancer efficacy of chemotherapeutic drugs to tumor cells, especially in the drug-resistant cells. Thus, ivermectin, a FDA-approved antiparasitic drug, could potentially be used in combination with chemotherapeutic agents to treat cancers and in particular, the drug-resistant cancers.

**Electronic supplementary material:**

The online version of this article (10.1186/s13046-019-1251-7) contains supplementary material, which is available to authorized users.

## Background

The development of multidrug resistance (MDR) is detrimental to successful chemotherapy in various cancers [1–3]. There are various mechanisms underlying the resistance to chemotherapeutic drugs in cancer cells [3–6]. A major mechanism of resistance is the overexpression of ATP-binding cassette (ABC) transporters, which could efflux the chemotherapeutic agents out of cells [7–9]. P-glycoprotein (P-gp), also known as multidrug resistance protein 1 (MDR1) or ABCB1, was the first discovered and best studied ABC transporter [4, 10].

Multidrug resistance reversal agents, also known as MDR regulators/modulators or chemotherapy sensitizers, have been found to ameliorate the drug resistance in cancer cells *in vitro* and in animal models *in vivo* [5, 11, 12]. However, these agents have failed to demonstrate satisfactory efficacy in clinical trials due to the poor reversal efficacy, excessive toxicity, or interference with the pharmacokinetics of chemotherapeutic drugs [5, 12–14]. Therefore, it is urgently needed to develop novel MDR reversal agents that could be further used clinically for the treatment of the resistant cancers.

Avermectins, a class of 16-membered macrolide compounds, are widely used to treat parasites and pest insects [15]. Ivermectin (IVM), an avermectin derivative, was found to be especially effective against a variety of parasites and disease vectors that could be used in humans [16–18]. Recently, IVM has been found to inhibit the growth of some human cancer cells [19, 20]. In addition, IVM was also found to inhibit the ATPase activity of P-gp [21, 22] and reverse the P-gp-related multidrug resistance *in vitro* [21, 23, 24]. However, the detailed underlying mechanisms of how IVM enhances the sensitivity of the cells to the chemotherapeutic agents and reverses the resistance of the tumor cells remain largely unknown. And whether IVM could reverse the multidrug resistance *in vivo* has not been elucidated.

In this study, we used multiple tumor cell lines, including vincristine (VCR)-sensitive/resistant HCT-8 colorectal cancer cells, adriamycin (ADR)-sensitive/resistant MCF-7 breast adenocarcinoma cells and ADR-sensitive/resistant K562 chronic myeloid leukemia cells, as well as two xenograft tumor models, to investigate whether IVM could reverse the drug resistance of cancer cells. These cancer cell lines were used because both colorectal cancer and breast adenocarcinoma are among the most common malignant solid tumors [25, 26], and chronic myeloid leukemia (CML) is one of the most common malignant hematological neoplasms [27]. In this study, we found that IVM could increase the sensitivity of the cancer cells and, in particular, the resistant cancer cells to the chemotherapeutic drugs and even reverse the resistance of the cancer cells to the drugs both *in vitro* and *in vivo*, and we identified a novel molecular mechanism underlying the reversal of the chemotherapeutic drug-resistance by IVM in cancer cells.

## Methods

### Cell viability analysis

VCR-sensitive/resistant human colorectal cancer cell line HCT-8, ADR-sensitive/resistant human breast adenocarcinoma cell line MCF-7 (both from Huiying BioTech), ADR-sensitive/resistant human chronic myelogenous leukemia cell line K562, human colorectal cancer cell line HCT-116 (both from KeyGen Biotech), and EGFR knockout HCT-116 cell line [28] [kindly provided by Dr. Ningzhi Xu at Chinese Academy of Medical Sciences] were maintained in RPMI-1640 medium (Sigma-Aldrich), supplemented with 10% FBS at 37°C in a humidified atmosphere with 5% CO_2._ Different concentrations of IVM (Meilun BioTech), VCR (YuanchengGongchuang Tech), ADR (KeyGen Biotech) or mitomycin C (Welson Biotech) were used to treat the cells. After 48 h incubation, cells were subjected to MTT analysis and the absorbance at 570 nm was recorded by a Spectra Max i3 microplate reader (Molecular Devices Corp., Sunnyvale, CA, USA).

### Xenograft models in mice

A xenograft colorectal carcinoma mouse model was established by injecting 1×10^7^ VCR-sensitive or resistant HCT-8 cells subcutaneously in the flank region of each female nude BALB/c mice (4-week-old, Vital River Lab). When tumors reached about 100 mm^3^, the nude mice were randomized into four groups (n = 6) according to tumor volumes and body weights. Drugs were injected intraperitoneally daily for 27 days, including IVM (2 mg/kg/day), VCR (0.2 mg/kg/day), IVM (2 mg/kg/day) plus VCR (0.2 mg/kg/day). To prepare IVM for injection, a stock solution (5 mg/ml in DMSO) was prepared and then diluted by using 0.9% NaCl, which resulted in a homogeneous suspension of IVM. Two hundred microliters (200 μl) of the IVM was injected to each mouse. VCR was also prepared in 0.9% NaCl, and mice injected with only 0.9% NaCl solution served as vehicle control. Tumor volume was measured every three days by using calipers. Tumor volumes were calculated as V = length × width^2^/2. On the 27^th^ day, the tumors were harvested, weighed, and then fixed in 4% paraformaldehyde for immunofluorescence and immunohistochemistry analysis.

In order to establish the leukemia mouse model with K562 cells, the male non-obese diabetic/severe combined immune deficient NOD/SCID mice (4-week-old, Vital River) were given cyclophosphamide (Meilun BioTech) (2 mg/mouse/day) for three days before the ADR-sensitive/resistant K562 cells were injected (2 ×10^7^ cells/mouse) into tail vein. Then, the mice were randomized into three groups (n = 6). The drugs ADR (0.3 mg/kg/day) and/or IVM (2 mg/kg/day) were injected intraperitoneally daily for 27 days. All of the drugs were prepared in 0.9% NaCl and mice injected with only 0.9% NaCl solution served as vehicle control. On the 27^th^ day, the mice were sacrificed, and spleen was weighed, and then fixed in 4% paraformaldehyde for histopathological examination. The peripheral blood was collected in anticoagulant heparin. Cells within bone marrow were washed out by 10 mM phosphate-buffered saline (PBS, pH 7.4). Blood smears were prepared and stained with May-Grünwald Giemsa (MGG) staining. The peripheral blood cells and bone marrow cells were subjected to flow cytometry after stained with mouse anti-human CD33-FITC (555626), CD13-PE (555748), and isotype-matched FITC- (555394), PE- (555749) conjugated control antibodies (all from BD Biosciences).

### HPLC analysis of VCR

One milliliter of distilled water was added to the cell pellets and the cells were subjected to freezing (at -80°C) and thawing for three times. The tumor tissues were homogenized with 600 μl of H_2_SO_4_ in a glass homogenizer on ice. After centrifugation, the supernatants were dried by vacuum, and then resuspended in 200 μl of distilled water, and analyzed by an Agilent 1100 series HPLC system (California, USA). The samples were injected into the C18 column (250 mm × 4.6 mm, 5 μm) with the mobile phase containing 20 mM KH_2_PO_4_ (pH 6.6) and methanol (30:70, v/v). The detection wavelength was 298 nm.

### Western blotting analysis

Cells or tumor tissues were homogenized in the buffer containing 50 mM Tris-HCl with pH 7.5, 150 mM NaCl, 1% Triton X-100, 1 mM EDTA, 1 mM PMSF and 1% protease inhibitors. Lysates were centrifuged at 5, 000 × *g* for 15 min at 4°C and the loading buffer was added to the supernatants. The protein samples were boiled at 100°C for 10 min and electrophoresed in SDS-polyacrylamide gels. Then the gels were transferred onto PVDF membranes (Millipore, Darmstadt, Germany). The membranes were blocked in 5% bovine serum albumin (BSA) (w/v) or 5% fat-free milk (w/v) in Tris-buffered saline with 0.1% Tween 20 (TBST) buffer for 2 h at RT, incubated with the corresponding antibody at 4°C overnight, then incubated with the horseradish peroxidase (HRP)-labelled secondary antibody for 3 h at RT. The following antibodies were used: anti-EGFR (#2232, 1:1000), anti-p-EGFR (#2234, 1:500), anti-P65 (#8242, 1:1000), anti-p-P65 (#3033, 1:500), anti-p-Akt (#9271, 1:500), anti-p-ERK (#4370, 1:500), anti-Akt (#9272, 1:1000), and anti-ERK (#9102, 1:1000) (All from Cell Signaling); anti-P-gp (517310, 1:500, Calbiochem) and anti-GAPDH (CW0100, 1:1000, Beijing Com Win). Finally, the membranes were stained with standard ECL reagents and then photographs were taken by DNR MicroChemi4.2 system (Bio-Imaging Systems Ltd, Neve Yamin, Israel).

### Quantitative PCR analysis

HCT-8 cell pellets, mouse peripheral blood cells and mouse bone marrow cells were suspended respectively and homogenized in 1 ml Trizol reagent (Invitrogen, Carlsbad, CA, USA) on ice. Then, the mixture was placed at RT for 5 min. Two hundred microliters of chloroform were added. The tubes were fiercely shaken for 1 min and centrifuged at 12, 000 × *g* for 15 min at 4°C. Then the supernatant was transferred into a new centrifuge tube, and 500 μl of propanol was added. The tubes were fiercely shaken for 1 min and centrifuged at 12, 000 × *g* for 10 min at 4°C. The precipitant was washed with 75% ethanol twice, dried and dissolved in RNase free ddH_2_O. The total RNA concentration was measured using Biophotometer Plus (Eppendorf, Hamburg, Germany). Total RNA (0.3 ~ 1 μg) was reverse-transcribed into cDNA by using a M-MuLV reverse transcriptase assay kit (Fermentas, Ontario, Canada). The relative mRNA levels of MDR1 and bcr/abl fusion gene were determined by quantitative PCR using a SYBR green Premix Ex Taq^TM^ (Tli RNaseH Plus) PCR kit (TaKaRa, Dalian, China) in a MX3000P real-time thermocycler (Axygen, California, USA). The primer sequences for MDR1 were 5′-GACATGACCAGGTATGCCTA-3′ (sense) and 5′-CTTGGAGACATCATCTGTAAGTC-3′ (antisense); the primer sequences for bcr/abl fusion gene were 5′-GGAGCTGCAGATGCTGACCAAC-3′ (sense) and 5′-TCAGACCCTGAGGCTCAAAGTC-3′ (antisense) and the primer sequences for the control gene GAPDH were 5′-CGCTGAGTACGTCGTGGAGTC-3′ (sense) and 5′-GCTGATGATCTTGAGGCTGTTGTC-3′ (antisense).

### Luciferase reporter assay

A 1, 637 bp region encompassing the NF-κB binding site and the annotated transcription start of ABCB1 (-1468 to +168 bp, chr7-: 87713155-87714791) was cloned into a Gaussia luciferase (GLuc) reporter vector (pEZX-PG04, Genecopoeia), which contains a reference reporter gene, secreted alkaline phosphatase (SeAP).

The cells in 24-well plates were co-transfected with the above reporter vector with pcDNA3.1(+)-P65 expression vector or siRNA targeting NF-κB using transfection reagent VigoFect (Vigorous Biotech). After 12 h, the cells were treated with 3 μM IVM and/or 25 nM VCR for 48 h. The activities of GLuc and SeAP were quantified with the secrete-pair dual luminescence assay kit (Genecopoeia).

### Immunohistochemistry and immunofluorescence analysis

The fixed tumor tissues in nude mice were frozen and cut into 5 μm thick sections. The sections were fixed in 4% paraformaldehyde for 10 min at RT, perforated by 0.5% Triton-X-100 for 10 min at RT. Then endogenous peroxides were removed, and sections were blocked in TBST containing 3% BSA for 1 h at RT, incubated with anti-P-gp antibody overnight at 4°C and HRP-labelled secondary antibody for 2 h at RT. Then, immunoreactive sites were subsequently identified by using the 3,3′-diaminobenzidine (DAB) substrate kit (Vector Laboratories). The nuclei were stained with hematoxylin for 3 min at RT and the frozen sections were visualized under Olympus IX71 inverted microscope (Tokyo, Japan). For the immunofluorescence analysis, the processes of fixation, perforation and blocking were the same as those of immunohistochemistry. Then slides were incubated with the anti-P-gp antibody at 37°C for 1 h and FITC-labeled secondary antibody for 1 h at 37°C. All images were acquired using a Carl Zeiss LSM710 laser scanning confocal microscope (Oberkochen, Germany).

### Flow cytometry

Two hundred microliters of peripheral anticoagulant heparin-treated blood and 1×10^6^ bone marrow cells were treated using red-blood-cell lysing buffer (BD Biosciences). White blood cells in peripheral blood and bone marrow cells were resuspended in 100 μl of PBS, incubated with 20 μl of mouse anti-human antibodies, which include CD13-PE (555394), CD33-FITC (555626), and isotype-matched FITC- (555748), PE- (555749) conjugated control antibodies (all from BD Biosciences), for 30 min at 4°C. The cells were washed with PBS and resuspended in 300 μl of 2% paraformaldehyde and detected by FACS Aria II flow cytometry (Becton Dickinson, USA).

### Staining

Ten microliters of anticoagulant blood were smeared on each glass microscope slide. Then, the slides of blood cells smears were dried and fixed in methanol for 5 min at RT, and then immersed into May-Grünwald solution for 3 min, then immersed into PBS solution (pH 6.8) for 1 min. Finally, the slides were stained with Giemsa solution (diluted 20 times with the PBS) for 10 min and washed with ddH_2_O for 30 s, air-dried and visualized under Olympus IX71 inverted microscope (Tokyo, Japan).

After the NOD/SCID mice were sacrificed, the spleen was harvested and fixed with 4% paraformaldehyde. Then these tissues were dehydrated in a series of alcohol, embedded in paraffin and sliced into 5-μm sections. Hematoxylin and eosin staining was carried out according to the routine staining method. Briefly, the sections were dewaxed, rehydrated in alcohol, stained with hematoxylin for 15 min, differentiated, and then stained with eosin for 3 min, dehydrated in alcohol and xylene, and mounted. The sections were examined under Olympus IX71 inverted microscope (Tokyo, Japan).

### Co-immunoprecipitation (Co-IP) assay

The cells were lysed and then centrifuged. The supernatants were incubated with the anti-avermectins (AVMs) antibody, which had a cross-reactivity of 100% with abamectin (ABM) and 25% with IVM [29] (provided by Dr. Jianzhong Shen) at 4°C in rotation overnight. Then 80 μl of protein G plus A agarose (Beyotime Biotechnology, Jiangsu, China) was added and the mixture was incubated at 4°C in rotation for another 6 h. Then, the immunocomplexes were washed and the precipitated beads were resuspended in 50 μl of 2 × loading buffer for the electrophoresis.

### Chromatin immunoprecipitation assay

Chromatin immunoprecipitation (ChIP) was performed using the EZ ChIP kit (EMD Millipore). Briefly, HCT-8 cells treated with IVM for 48 h were collected and cross-linked with formaldehyde. Chromatin was sonicated and then incubated and precipitated with anti-P65, anti-RNA polymerase II (positive control), or normal rabbit IgG (negative control), respectively. The immunoprecipitated DNA fragments were detected by qPCR analysis. The primers for the MDR1 promoter (-1468 to -1319 bp) were 5'-AAACGGATGCATGGGGCGG-3' (sense) and 5'-GAAGATAGACAACTGGTTAGACGAG-3' (antisense).

### Plasmids, siRNA and adenovirus

Human full length MDR1 (AF016535.1), human full length EGFR (NM_005228.4) and human full length RELA/P65 (NM_021975.3) were cloned into pcDNA3.1(+) vector (GENEWIZ). HCT-8 cells were transfected using the transfection reagent VigoFect. pGenesil-P-gp vector was used to express shRNA of P-gp in the cells [30]. Three siRNAs targeting EGFR and NF-κB (P65) were synthesized by Shanghai Gene Pharma Co. Ltd (Shanghai, China). The siRNA with the highest gene silencing efficacy was chosen for further use. The recombinant adenoviral vectors expressing LacZ (Ad-LacZ), Akt (Ad-Akt-myr), MKK1 (Ad-MKK1-R4F) or mTOR (Ad-mTOR) (all provided by Dr. Shile Huang at Louisiana State University), were amplified and used as described in the reference [31] to constitutively activate Akt, ERK and mTOR, respectively. Ad-MKK1-R4F was used for the activation of ERK because MKK1 could phosphorylate and activate ERK in the cells.

### Affinity determination

The interactions between IVM and EGFR extracellular domain were determined using Super Streptavidin (SSA) biosensors in the Octet RED96 system (ForteBio Inc., Menlo Park, CA, USA). First, the recombinant extracellular domain of human EGFR protein (ab155639, Abcam) was biotinylated and loaded onto the SSA biosensors at 40 μg/mL in PBS containing 0.05% Tween-20 and 0.1% BSA. The biosensors were blocked with biocytin (5 μg/ml) for 60 s. Diluted IVM in PBS solution containing 0.05% Tween-20, 0.1% BSA and 10% DMSO was then added onto the SSA biosensors loaded with EGFR extracellular domain. The real time binding response (Δλ in nanometer, nm) between IVM and EGFR was calculated by subtracting the nonspecific binding of IVM to the SSA biosensors from the binding of IVM with EGFR. The kinetic parameters and affinities were calculated with a non-linear global fit of the data, using Octet data analysis software version 8.5 (ForteBio Inc., Menlo Park, CA, USA)

### Statistics

All experiments were repeated at least three times except that some WB experiments were repeated twice. Statistical significances for survival percentage results in NOD/SCID mice were determined using the log-rank test. In other cases, a one-way analysis of variance (ANOVA) followed by Dunnett’s test was used for multiple comparisons. Values of *P* < 0.05 were considered significant, and values of *P* < 0.01 were considered extremely significant. All data are mean ± SD unless otherwise indicated.

## Results

### Ivermectin reverses the resistance of tumor cells to chemotherapeutic drugs

**Fig. 1:**
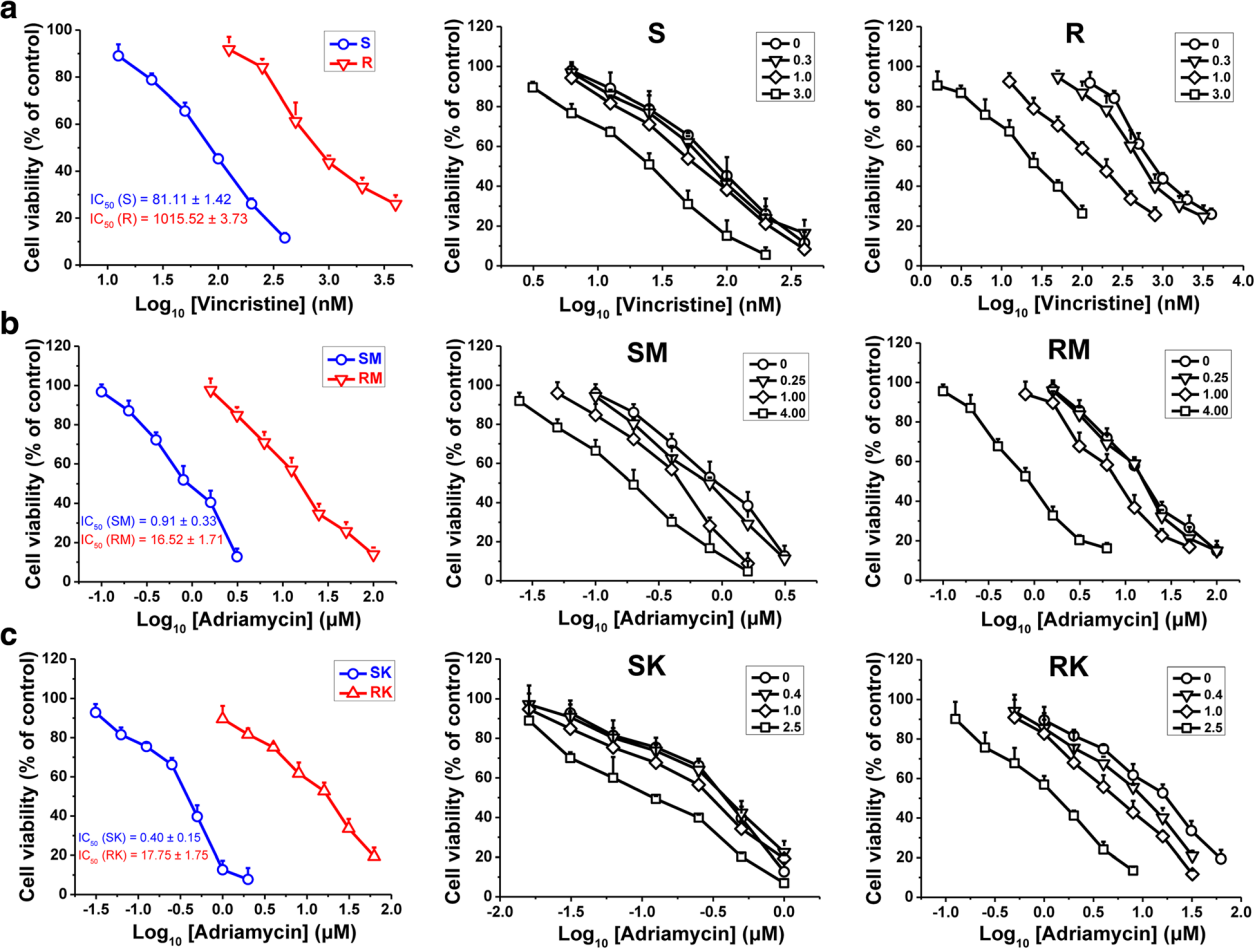
Ivermectin increased the sensitivity of the cells to chemotherapeutic drugs. **a-c** The cell viability of sensitive or resistant HCT-8 cells (**a**), MCF-7 cells (**b**) and K562 cells (**c**) after treated with vincristine or adriamycin with or without different concentrations of ivermectin (IVM) for 48 h. Cell viability was determined by MTT assay. The numbers in the figure keys represent the concentrations (μM) of IVM. Cells treated with vehicle serve as a blank control. Abbreviations: IVM, ivermectin; S, vincristine-sensitive HCT-8 cells; R, vincristine-resistant HCT-8 cells; SM, adriamycin-sensitive MCF-7 cells; RM, adriamycin-resistant MCF-7 cells; SK, adriamycin-sensitive K562 cells; RK, adriamycin-resistant K562 cells. All experiments were conducted in quintuplicates and data were expressed as the mean ± SD (n = 5)

We first assessed the effect of ivermectin (IVM) on VCR-sensitive/resistant HCT-8 human colorectal cancer cells. As shown in Fig. 1a (left panel), the IC_50_ value of VCR in the resistant cells (R cells) was ten times more than that in the sensitive cells (S cells). However, for IVM, the IC_50_ values were quite close in the S and R cells (Additional file 1: Figure S1A). Furthermore, we found that IVM increased the sensitivity of the cells to VCR in a dose-dependent manner in R cells (Fig. 1a, right panel); however, only high concentration of IVM can increase the sensitivity of the cells to the drug in the S cells (Fig. 1a, middle panel).

We then sought to determine whether IVM had a similar effect on other cancer cells, such as human breast cancer cell line MCF-7 and human chronic myelogenous leukemia cell line K562. Consistently, in the presence of IVM, the IC_50_ values (μM) of ADR in the ADR-sensitive MCF-7 and K562 cells (simplified as SM and SK cells respectively), decreased from 0.91 and 0.40 to 0.21 and 0.11, respectively, while the IC_50_ values (μM) in the ADR-resistant MCF-7 and K562 cells (simplified as RM and RK cells respectively) decreased from 16.52 and 17.75 to 1.02 and 1.23, respectively (Fig. 1b and c; Additional file 1: Figure S1A). Thus, IVM treatment obviously increased the sensitivity of MCF-7 and K562 cells to ADR. Altogether, IVM reversed the resistance of multiple cell lines to the chemotherapeutic agents.

Furthermore, we tested whether IVM could reverse the resistance to other chemotherapeutic drugs. We found that the cancer cells became more sensitive to two other chemotherapeutic drugs mitomycin C (MC) and adriamycin (ADR), when the cells were treated with IVM (Additional file 1: Figure S1B & C). The “sensitivity ratio”, which represents the change of the sensitivity of the cells to a chemotherapeutic drug after the cells were treated by a chemical, was calculated as the ratio of the IC_50_ value of the drug against the cells without the IVM treatment over that with IVM treatment (Table 1). The sensitivity ratios of the three drugs VCR, MC, and ADR in the R cells were about 11, 3, and 4 times of those in the S cells, respectively. This result not only indicated that the cells with resistance to VCR had cross-resistance to the other drugs MC and ADR, but also demonstrated that IVM enhanced the effects of chemotherapeutic drugs in both S and R cells, and the effects of IVM were much stronger in the R cells than in the S cells. The above results indicated that IVM increased the sensitivity of the cells to the particular chemotherapeutic agents.

**Table 1:** Change of sensitivity of the HCT-8 cells to chemotherapeutic drugs after the treatment of 3 μM ivermectin

Test drugs	IC_50_ (μM)				Sensitivity ratios	
	S		R		[IC_50_ (- IVM)/IC_50_ (+ IVM)]	
	- IVM	+ IVM	- IVM	+ IVM	S	R
Vincristine	0.081 ± 0.001	0.027 ± 0.001	1.015 ± 0.003	0.032 ± 0.001	2.96	31.15
Mitomycin C	2.71 ± 0.32	1.34 ± 0.22	26.62 ± 0.51	4.81 ± 0.32	2.02	5.53
Adriamycin	5.42 ± 0.34	0.42 ± 0.12	36.34 ± 0.93	0.74 ± 0.14	12.90	49.11

*Abbreviations*: S, vincristine-sensitive HCT-8 cells; R, vincristine-resistant HCT-8 cells; IVM, ivermectin

### Ivermectin significantly enhances the anti-tumor effect of vincristine in solid tumor xenograft

**Fig. 2:**
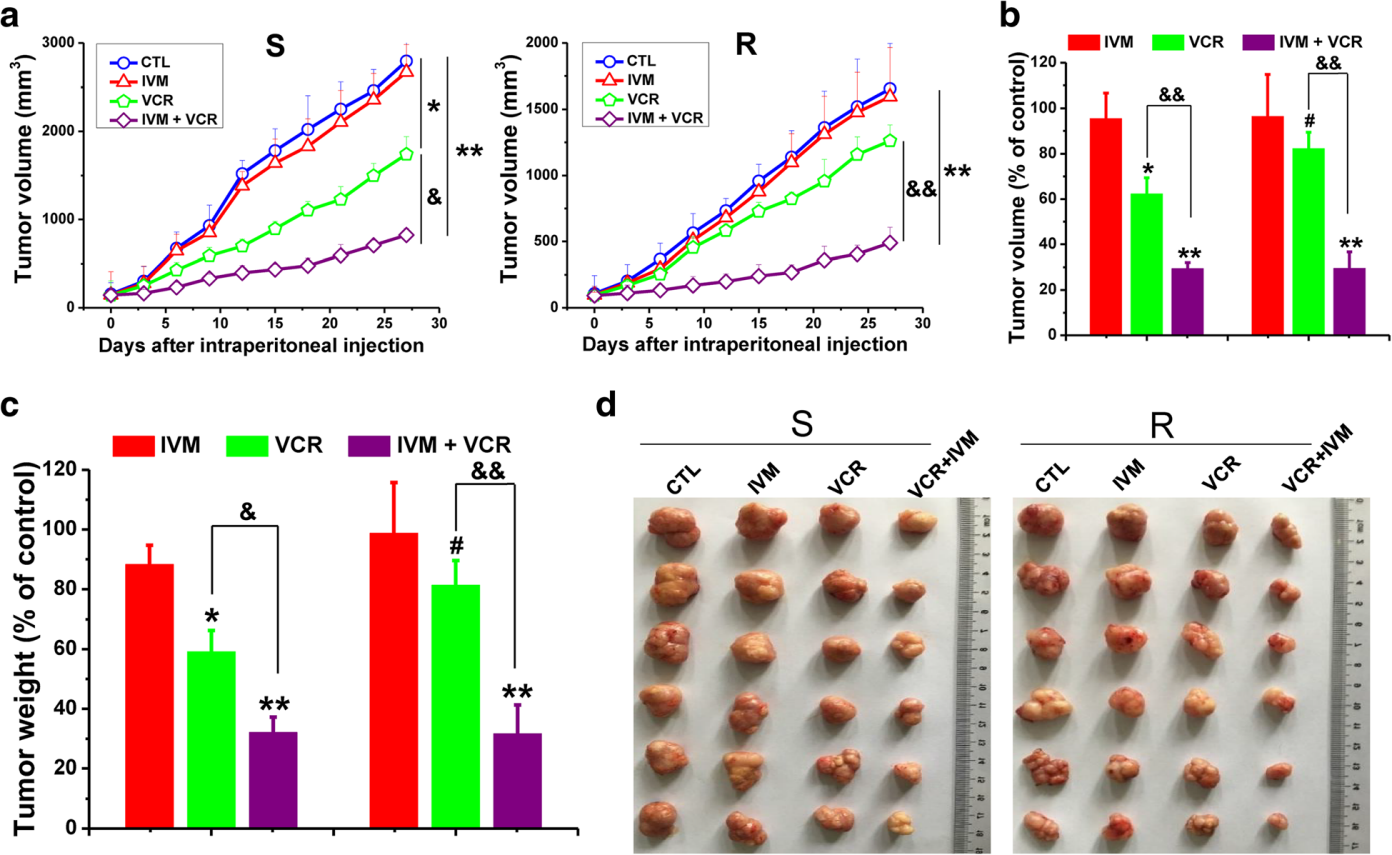
Ivermectin enhances the anti-tumor effect of vincristine in solid tumor xenografts. The nude mice were injected subcutaneously with 1×10^7^ HCT-8 cells, which are sensitive or resistant to vincristine (VCR). When the tumor reached to about 100 mm^3^, the mice were treated with ivermectin (IVM) (2 mg/kg) and/or VCR (0.2 mg/kg) by intraperitoneal injection daily for 27 days. **a** Changes of tumor volumes from day 0 to day 27; **b-d** Volumes (**b**), weights (**c**) and images (**d**) of the tumors on day 27. Mice treated with vehicle serve as control. The weights and volumes of the tumors in the control xenografts were 1.97 ± 0.12 g and 2794.5 ± 384.8 mm^3^ (in S group) vs 1.12 ± 0.11 g and 1654.8 ± 342.6 mm^3^ (in R group), respectively. Abbreviations: CTL, control; IVM, ivermectin; VCR, vincristine; S, vincristine-sensitive HCT-8 xenograft; R, vincristine-resistant HCT-8 xenograft. Data in **a**-**c** represent the mean ± SD (n = 6 mice each group). Statistical significances were determined using one-way ANOVA followed by Dunnett’s test. ^*^P < 0.05, ^**^P < 0.01, compared with the respective vehicle controls (blue columns/lines); ^#^*P* < 0.05, compared with the corresponding columns with the same color in the S group; ^&^*P* < 0.05, ^&&^*P* < 0.01, comparison between the two columns or lines

To evaluate whether IVM can suppress tumorigenesis *in vivo*, we used a human tumor xenograft model by subcutaneously injecting VCR-sensitive (S) or resistant (R) HCT-8 cells into the dorsal flank of female nude mice. The inhibitory effect of VCR on the growth of the tumors derived from R cells (simplified as R tumors) was weaker than that of the tumors derived from S cells (simplified as S tumors) (Fig. 2a and b). And after treated with VCR plus IVM, the growth rate of the S and R tumors was reduced compared with those with VCR alone treatment (Fig. 2a and b). In addition, the tumor weight (Fig. 2c) and the tumor size (Fig. 2d) from the mice treated with VCR plus IVM was much lower or smaller than those of the mice treated with VCR alone. These results indicated that IVM not only significantly reversed the resistance of R tumors to VCR but also strongly ameliorated the response of S tumors to VCR *in vivo*.

### Ivermectin enhances the anti-tumor effect of adriamycin in a mice model for human leukemia

**Fig. 3:**
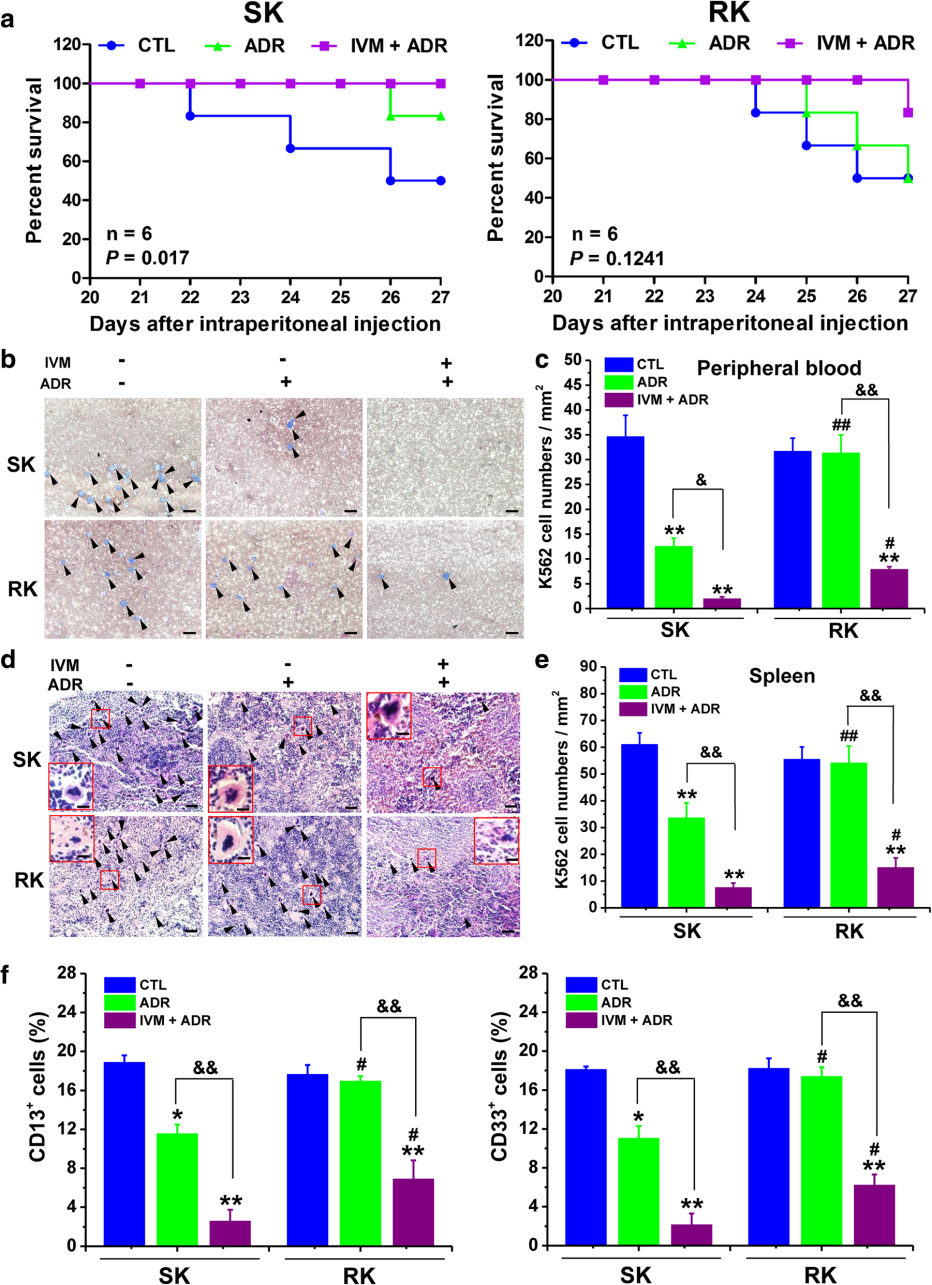
Ivermectin enhances the anti-cancer effect of adriamycin in a mice model for human leukemia. The NOD/SCID mice were injected through tail vein with 2×10^7^ K562 cells, which are sensitive or resistant to adriamycin (ADR). Then, the mice were treated with ADR (0.3 mg/kg, i.p.) alone or combined with ivermectin (IVM) (2 mg/kg, i.p.) daily for 27 days. **a** Survival percentage of the mice were calculated. **b** and **c** The May-Grünwald Giemsa (MGG) staining (**b**) and the K562 cell numbers (**c**) of the peripheral blood smear from the mice were determined. Scale bars: 150 μm. **d** The histopathological examination of spleen with hematoxylin and eosin (H & E) staining. Scale bars: 150 μm. The arrowheads indicate the K562 cells. The images within the red rectangles were enlarged as insets (scale bars: 30 μm). **e** The K562 cell numbers in spleen were determined based on the spleen H & E staining results. **f** The percentage of cells stained positive for cell surface markers CD13 or CD33 in bone marrow were determined by flow cytometry. Abbreviations: CTL, control; SK, adriamycin-sensitive K562 xenograft; RK, adriamycin-resistant K562 xenograft. Data represent the mean ± SD (n = 6). Statistical significances in **a** were determined by using the log-rank test. Statistical significances in **c**, **e**, and **f** were determined by using one-way ANOVA followed by Dunnett’s test. ^*^P < 0.05, ^**^P < 0.01, compared with the respective vehicle controls (blue columns); ^#^*P* < 0.05, ^##^*P* < 0.01, compared with the corresponding columns with the same color in the SK group; ^&^*P* < 0.05, ^&&^*P* < 0.01, comparison between the two columns (purple column vs green column)

To determine whether IVM can suppress tumorigenesis of non-solid tumor e.g. leukemia *in vivo*, we established a human tumor xenograft model by injecting ADR-sensitive (SK)/resistant (RK) K562 cells into the peripheral blood of male NOD/SCID mice via tail vein. The survival curves indicated that the survival percentage of the SK mice treated with ADR plus IVM was higher than that of the mice treated with vehicle. Although there was no statistical significance between RK mice treated with ADR plus IVM and those treated with vehicle, the overall survival percentage was higher in ADR plus IVM treatment group in RK mice (Fig. 3a). The body weight of the mice in ADR plus IVM treatment groups had almost no significant change throughout the experiment, while the body weight severely declined in the vehicle group (Additional file 1: Figure S2A), and the relative weight of spleen was almost restored to the normal level by IVM plus ADR treatments (Additional file 1: Figure S2B). These findings indicated that the RK leukemia was indeed resistant to ADR treatment, and co-treatment with IVM significantly enhanced the anticancer activity of ADR to both SK and RK leukemia.

In addition, we found that ADR alone treatment significantly decreased the K562 cell numbers in peripheral blood and spleen only in the mice with SK leukemia, but not RK leukemia (Fig. 3b-e). However, IVM plus ADR treatment decreased the number of K562 cells in both peripheral blood and spleen compared with the vehicle or ADR alone treatment in not only the mice with SK leukemia but also those with RK leukemia (Fig. 3b-e). Moreover, the cells with positive staining of CD13 or CD33, the surface markers of K562 cells, as well as the mRNA levels of bcr/abl fusion gene, a marker of chronic myeloid leukemia, in peripheral blood and bone marrow of IVM plus ADR-treated mice were lower than that in the ADR alone-treated mice (Fig. 3f; Additional file 1: Figure S2C & D). The above results indicated that IVM enhanced the anti-tumor effect of ADR in leukemia, and drastically reversed the resistance of leukemia to ADR *in vivo*.

### Ivermectin reverses the resistance by inhibiting P-gp expression

**Fig. 4:**
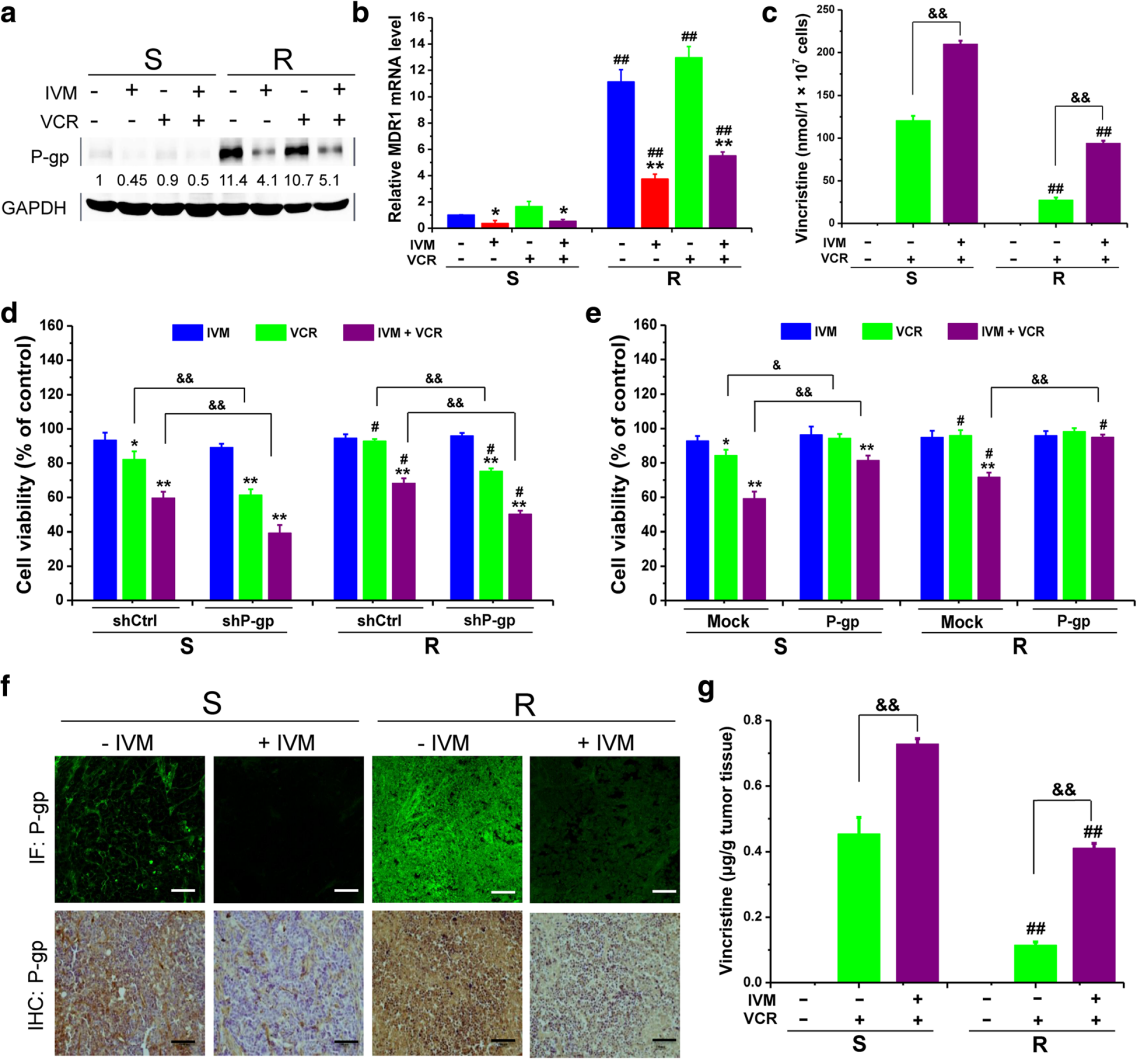
Ivermectin inhibited P-gp expression and increased the intracellular drug accumulation. **a-c** Protein level of P-gp (**a)**, mRNA level of MDR1 (**b**), or intracellular VCR concentrations (**c**) in HCT-8 cells treated with 25 nM VCR and/or 3 μM ivermectin (IVM) for 48 h were determined. The protein level was detected by Western blotting analysis and mRNA level was determined by qPCR using GAPDH as the internal control, and intracellular VCR concentrations were determined by HPLC. **d** and **e** Cell viability of HCT-8 cells transfected with the plasmid pGenesil-P-gp (P-gp shRNA) (**d**) or the plasmid pcDNA3.1(+)-P-gp (**e**) and then treated with 25 nM VCR and/or 3 μM IVM for 48 h. Cell viability was detected by MTT assay. Cells transfected with control shRNA (shCtrl)/empty vector pcDNA3.1(+) (mock) or treated with vehicle serve as control. **f** The P-gp expression in the HCT-8 xenografts was detected by immunofluorescence (upper panel) and immunohistochemical staining (lower panel). Green: P-gp protein. Scale bars: 200 μm. **g** VCR accumulation in tumor tissues of the mice was determined by HPLC analysis. Abbreviations: IVM, ivermectin; VCR, vincristine; S, vincristine-sensitive HCT-8 cells/xenografts; R, vincristine-resistant HCT-8 cells/xenografts. Data in **a** is the representative of three independent experiments. Data in **b** and **c** represent the mean ± SD (n = 3). Data in **d** and **e** represent the mean ± SD (n = 5). Data in **g** represent the mean ± SD (n = 6 mice in each group). Statistical significances were determined using one-way ANOVA followed by Dunnett’s test. ^*^P < 0.05, ^**^P < 0.01, compared with the respective vechicle controls; ^#^*P* < 0.05, ^##^*P* < 0.01, compared with the corresponding columns with the same color in the S group; ^&^*P* < 0.05, ^&&^*P* < 0.01, comparison between the two columns

The IC_50_ value of VCR in the R cells with P-gp knocked-down (150.01 nM) was significantly lower than that in the R cells without P-gp knocked-down (1015.52 nM), which indicated that P-gp was essential for the multidrug resistance in the R cells (Additional file 1: Figure S3A & B; Fig. 1a). Thus, we then determined whether IVM altered P-gp expression in HCT-8 cells. The mRNA and protein levels of MDR1/P-gp in the R cells were indeed higher than that in the S cells, and mRNA and protein levels of MDR1/P-gp were decreased by IVM in both S and R cells (Fig. 4a and b). In addition, after treatment of VCR plus IVM, the intracellular level of VCR increased compared with that in the cells treated with VCR alone (Fig. 4c). Altogether, these results suggested that IVM inhibited the expression and function of P-gp.

Furthermore, the inhibited cell viability of both S and R cells by the chemicals treatment was further reduced in the P-gp knocked-down cells (Fig. 4d). The IVM-reduced P-gp expression was recovered by the overexpression of P-gp or the treatment of sulforaphane (SFP), an activator of the transcription factor Nrf2, which could induce the expression of P-gp [32] (Additional file 1: Figure S3C & D), and the viability of the cells treated with IVM plus VCR increased compared with that of the cells without P-gp overexpression or SFP treatment, and this effect was more obvious in the R cells than that in the S cells (Fig. 4e; Additional file 1: Figure S3E). Thus, these results indicated that P-gp overexpression played a very important role in VCR resistance, and IVM could increase the sensitivity of the cells to VCR by inhibiting P-gp expression.

Consistently, in the solid tumor xenograft model, by using IF and IHC methods, we also found that the P-gp expression in the S tumors was lower than that in the R tumors, and IVM inhibited the expression of P-gp (Fig. 4f). In addition, the VCR concentrations in both S tumor and R tumor tissues increased after IVM treatment (Fig. 4g), indicating that IVM could inhibit P-gp expression and function *in vivo*.

### Ivermectin reverses the resistance of cells to the drugs through the inhibition of EGFR/ERK/Akt/NF-κB pathway

**Fig. 5:**
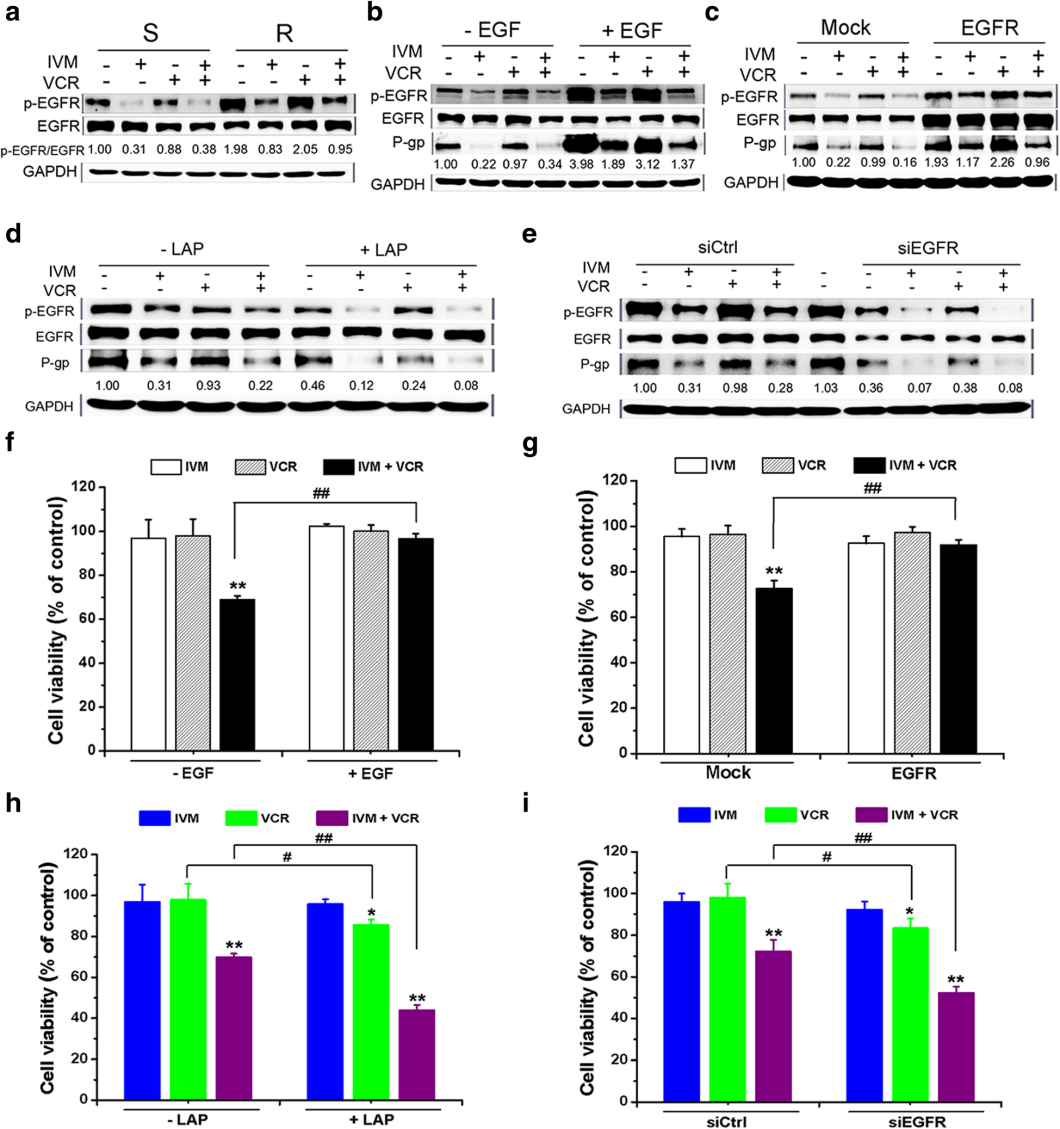
Ivermectin decreased P-gp expression by inhibiting the EGFR activation. **a** The expression levels of the proteins in the VCR-resistant/sensitive HCT-8 cells treated with 25 nM VCR and/or 3 μM IVM for 48 h were detected. **b-i** The expression levels of the proteins (**b**-**e**) and the cell viability (**f**-**i**) of the VCR-resistant HCT-8 cells untransfected (**b**, **d**, **f**, and **h**) or transfected with the plasmid pcDNA3.1(+)-EGFR (**c** and **g**) or siRNA for EGFR (**e** and **i**), treated with 25 nM VCR and/or 3 μM IVM in the presence or absence of 8 nM EGF (**b** and **f**) or 1 μM lapatinib (LAP), an EGFR inhibitor (**d** and **h**), for 48 h were determined. Cell viability was detected by MTT assay and the protein expression levels were detected by Western blotting analysis using GAPDH as internal control. Cells treated with vehicle, or transfected with empty vector pcDNA3.1(+) (mock)/control siRNA (siCtrl) serve as control. Abbreviations: EGF, epidermal growth factor; IVM, ivermectin; VCR, vincristine; S, vincristine-sensitive HCT-8 cells; R, vincristine-resistant HCT-8 cells. Data in **a**-**e** are the representative of two independent experiments. Data in **f**-**i** represent the percentage of respective control values (mean ± SD, n = 5). Statistical significances in **f**-**i** were determined using one-way ANOVA followed by Dunnett’s test. ^*^P < 0.05 and ^**^P < 0.01, compared with the respective controls; ^#^*P* < 0.05 and ^##^*P* < 0.01, comparison between the two columns

We then sought to reveal the underlying molecular mechanism of how IVM regulated P-gp expression and reversed MDR. It has been reported that P-gp expression could be regulated by EGFR signaling [33, 34]. Thus, we sought to determine whether EGFR pathway was involved in the regulation of the expression of P-gp and the sensitivity of cancer cells to VCR by IVM. In our experiments, we found that EGFR was over-activated in the HCT-8 R cells compared with the S cells. And the activation of these proteins were inhibited by the treatment of IVM alone or in combination with VCR (Fig. 5a).

Therefore, we then determined whether the effect of IVM on the P-gp expression was mediated by the activation of EGFR signal. The IVM-reduced P-gp level and EGFR activation were restored by EGF treatment or EGFR overexpression (Fig. 5b and c). On the other hand, treatment with an EGFR inhibitor lapatinib (LAP) or knockdown of EGFR by siRNA further reduced the IVM-suppressed P-gp level and the activation of EGFR (Fig. 5d and e). Hence, IVM could inhibit P-gp expression by inhibiting the activation of EGFR.

**Fig. 6:**
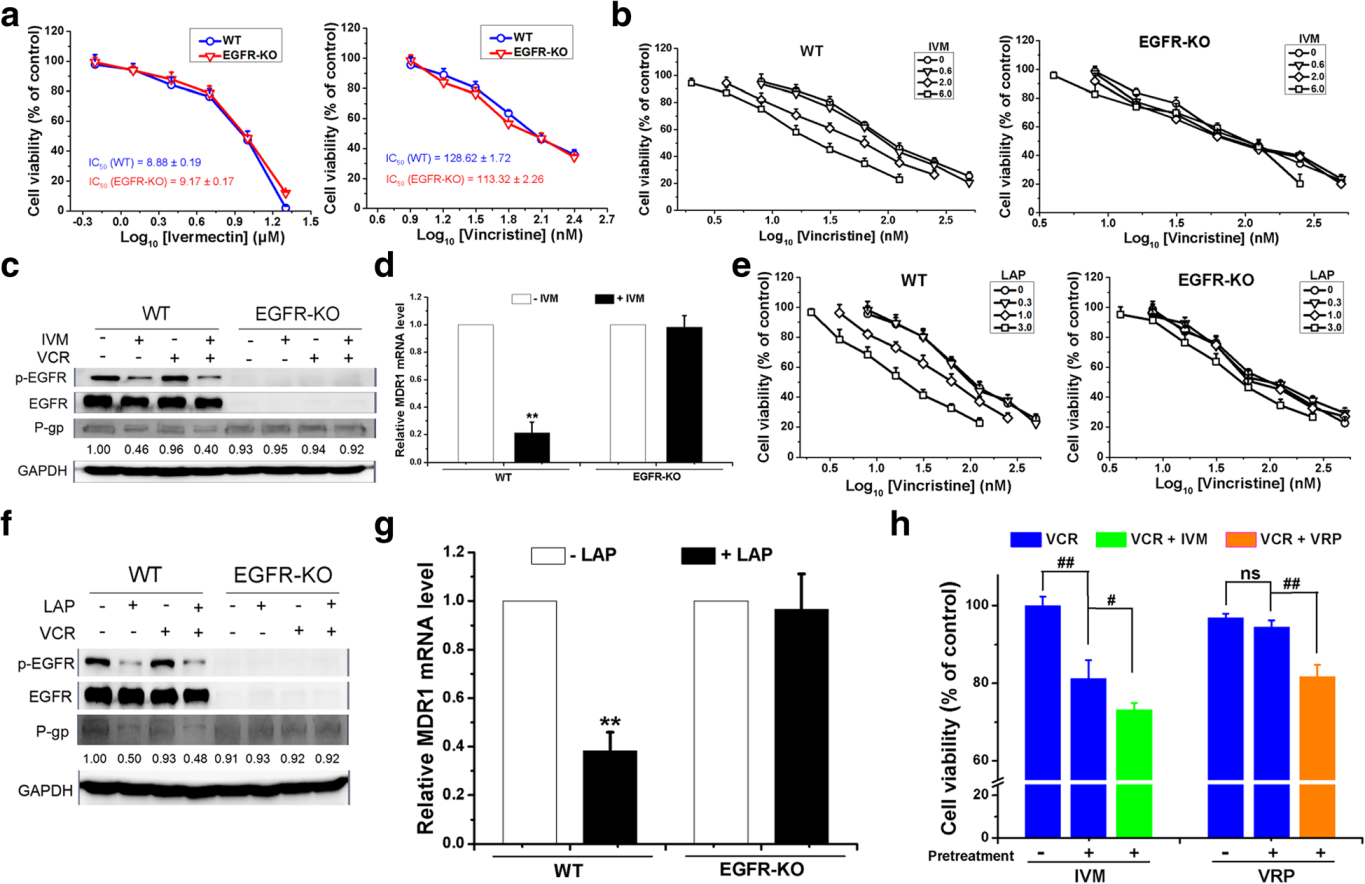
The effect of IVM on the EGFR signaling pathway. **a**-**g** The cell viability (**a**, **b,** and **e**), the protein expression levels of p-EGFR/EGFR and P-gp (**c** and **f**), and the mRNA level of MDR1 (**d** and **g**) of HCT-116 cells (WT) and EGFR knockout HCT-116 cells (EGFR-KO) treated with different concentrations of IVM or VCR (**a**), or treated with VCR in the presence of IVM (**b, c**) or LAP (**e, f**) or treated with IVM alone (**d**) or LAP alone (**g**) for 48 h were determined. The numbers in the figure keys in **b** and **e** represent the concentrations (μM) of IVM or LAP. **h** The cell viability of the VCR-resistant HCT-8 cells pretreated with IVM or VRP for 48 h, and then treated with VCR alone or VCR plus IVM, or VCR plus VRP for another 48 h were detected. Cell viability was determined by MTT assay and the protein expression levels were detected by Western blotting analysis using GAPDH as internal control. Cells treated with vehicle serve as control. Abbreviations: IVM, ivermectin; LAP, lapatinib; VCR, vincristine; VRP: verapamil; WT, HCT-116 cell; EGFR-KO, EGFR-knockout HCT-116 cells. Data in **a**, **b**, and **e** were conducted in quintuplicates and data were expressed as the mean ± SD (n = 5). Data in **c** and **f** are the representative of two independent experiments. Data in **d** and **g** are expressed as the mean ± SD (n = 3). Data in **h** represent the percentage of respective control values (mean ± SD, n = 5). Statistical significances in **d**, **g**, and **h** were determined using one-way ANOVA followed by Dunnett’s test. ^**^P < 0.01, compared with the respective vehicle controls; ^#^*P* < 0.05, ^##^*P* < 0.01, comparison between the two columns; ns, no significance (*P* > 0.05), comparison between the two columns

When the cells were treated with VCR plus IVM in the presence of EGF or with EGFR overexpression, the cell viability increased compared with the cells treated with VCR plus IVM in the absence of EGF or without EGFR overexpression (Fig. 5f and g), whereas the treatment of EGFR inhibitor LAP or knockdown of EGFR further decreased the cell viability, which was inhibited by VCR plus IVM treatment (Fig. 5h and i). Thus, IVM could increase the sensitivity of the cells to VCR by inhibiting EGFR. In order to prove that the reversal effects of IVM was indeed mediated by the inhibition of EGFR, we treated the HCT-116 cells and EGFR knockout HCT-116 cells with VCR and IVM. We found that the IC_50_ value of IVM and VCR was not significantly different between the wild type cells and the EGFR knockout cells (Fig. 6a). IVM increased the sensitivity of the HCT-116 cells to VCR in a dose-dependent manner, which was consistent with the results in HCT-8 cells; however, IVM could not increase the sensitivity of the EGFR knockout cells to VCR (Fig. 6b). As shown in Fig. 6c and d, the expression levels of the proteins p-EGFR and P-gp and the mRNA level of MDR1 were decreased by IVM alone or in combination with VCR in the wild type cells; while those were not altered by the treatment in the EGFR knockout cells. The similar result was observed in the cells with LAP treatment (Fig. 6e-g). Thus, the effect of IVM on P-gp expression was mainly mediated by EGFR. In addition, when HCT-8 cells were pretreated with IVM and then treated with VCR in presence or absence of IVM, the cells treated with VCR alone had lower cell viability compared with those cells treated with only VCR but without IVM pretreatment, which indicated that the reversal effect of IVM on the resistance of the cells to the drug still existed even after IVM was removed from the medium; however, pretreatment with VRP, a classical inhibitor of P-gp, did not change the viability of the cells treated with VCR alone, indicating that the reversal effect of VRP was present only when VRP was used at the same time with VCR (Fig. 6h). This result supported the notion that the reversal effect of IVM was not mediated by its direct inhibition of P-gp activity as the classical P-gp inhibitor VRP did. Altogether, the reversal effect of IVM on the resistance of the cells to the drug was largely mediated by the inhibition of EGFR phosphorylation, not by the direct inhibition of P-gp.

**Fig. 7:**
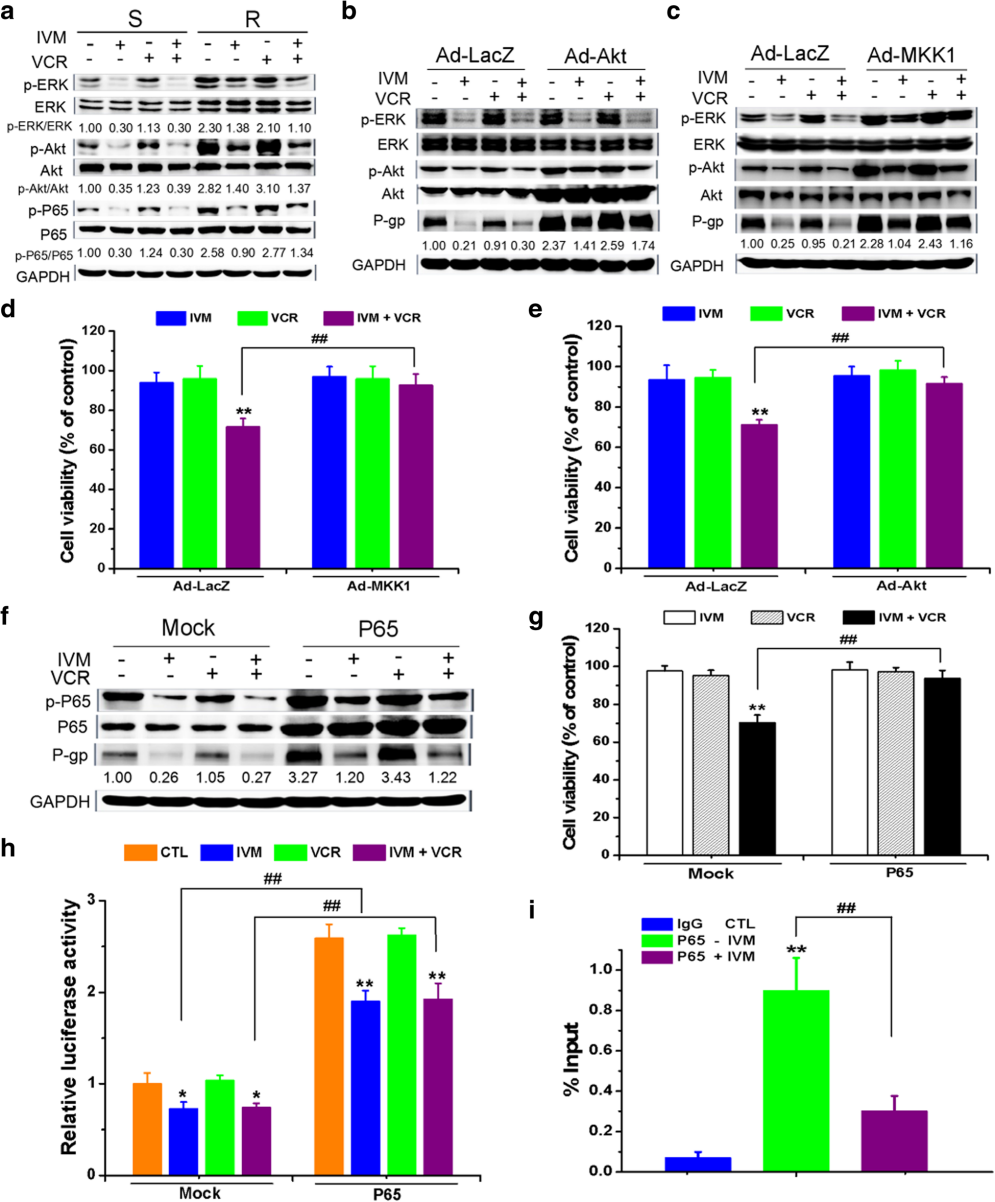
Ivermectin decreased P-gp expression through inhibiting ERK/Akt and NF-κB activation. **a** The expression levels of the proteins of the VCR-resistant/sensitive HCT-8 cells treated with 25 nM vincristine (VCR) and/or 3 μM ivermectin (IVM) for 48 h were determined. **b-h** Expression levels of the proteins (**b**, **c, f**), the cell viability (**d, e, g**), and the relative MDR1 promoter activity (**h**) of the VCR-resistant HCT-8 cells infected by recombinant adenovirus expressing HA-tagged constitutively active Akt (Ad-Akt-myr) (**b** and **e**) or by the flag-tagged constitutively active MKK1 (Ad-MKK1-R4F) (**c** and **d**), or transfected with plasmid pcDNA3.1(+)-P65, treated with 25 nM VCR and/or 3 μM IVM for 48 h were determined. **i** Chromatin IP was carried out with IgG (negative control) and anti-P65 antibody. Q-PCR result for MDR1 promoter region was shown as the percentage of input DNA. Cell viability was detected by MTT assay and the protein expression levels were detected by Western blotting analysis using GAPDH as internal control. Relative MDR1 promoter activity was determined by *Gaussia* luciferase activity normalized to the transfection control, i.e., secreted alkaline phosphatase (SeAP). Cells treated with recombinant adenovirus expressing Ad-LacZ or with empty vector pcDNA3.1(+) (mock) serve as control. Abbreviations: CTL, control; IVM, ivermectin; VCR, vincristine; S, vincristine-sensitive cells; R, vincristine-resistant cells. Western blots in **a**-**c** and **f** are representative of two independent experiments. Data in **d**, **e,** and **g** represent the percentage of respective control values (mean ± SD, n = 5). Data in **h** are expressed as fold change of the activity over the control from the mock group (mean ± SD, n = 3). Data in **i** are expressed as the mean ± SD (n = 4). Statistical significances in **d, e,** and **g-i** were determined using one-way ANOVA followed by Dunnett’s test. ^*^P < 0.05, ^**^P < 0.01, compared with the respective controls; ^##^*P* < 0.01, comparison between the two columns

We then sought to determine the downstream molecules of the inhibition of EGFR by IVM. We found that IVM inhibited ERK and Akt phosphorylation (Fig. 7a). Treatment with EGF or overexpression of EGFR stimulated the phosphorylation of ERK and Akt, which indicated that ERK and Akt were downstream of EGFR (Additional file 1: Figure S4A & B). Akt or ERK were constitutively activated by using adenoviral vectors Ad-Akt-myr and Ad-MKK1-R4F (MKK1 is the upstream kinase for ERK and could phosphorylate and stimulate ERK in the cells), respectively. Then, after the activation of Akt or ERK, the decrease of P-gp expression triggered by IVM was reduced (Fig. 7b and c), and the viability of the cells treated with VCR plus IVM was induced (Fig. 7d and e). In addition, treatment with a PI3K/Akt inhibitor wortmannin or an ERK inhibitor U0126 further inhibited the IVM-repressed P-gp expression and cell viability (Additional file 1: Figure S4C-F). Furthermore, we found that the ERK inhibitor U0126 also inhibited the activation of Akt, but the Akt inhibitor wortmannin did not inhibit ERK activation (Additional file 1: Figure S4G & H). Thus, ERK acted upstream of Akt. Indeed, activation of ERK by Ad-MKK1 also activated Akt, whereas activation of Akt by Ad-Akt did not activate ERK (Fig. 7b and c). In sum, these findings suggested that the increased sensitivity of cells to chemotherapeutic drugs by IVM was mediated by inhibiting the expression of P-gp via inhibiting the EGFR/ERK/Akt pathway.

It was known that NF-κB could be involved in the regulation of P-gp expression and the drug resistance in tumor cells [35]. In our studies, the level of phosphorylated P65 (p-P65), which could reflect the activation status of transcription factor NF-κB, was higher in the R cells than that in the S cells, and the level of p-P65 was significantly reduced after the cells were exposed to IVM (Fig. 7a). In addition, p-P65 level increased with treatment of EGF, overexpression of EGFR, or constitutively activation of Akt and ERK, indicating that NF-κB was downstream of EGFR/ERK/Akt (Additional file 1: Figure S5A-D). Furthermore, the down-regulation of p-P65 and P-gp by IVM were partially prevented by overexpression of P65 or treatment with an NF-κB activator phorbol-12-myristate-13-acetate (PMA) (Fig. 7f; Additional file 1: Figure S5E). And treatment with an NF-κB inhibitor PDTC or P65 knockdown further inhibited the IVM-repressed expression of P-gp and p-P65 (Additional file 1: Figure S5F & G). Thus, IVM inhibited the activation of NF-κB, which led to the reduced expression of P-gp. Upon treatment with VCR plus IVM, the cell viability increased in the P65-overexpressed cells or PMA-treated cells compared with the cells without P65 overexpression or PMA treatment (Fig. 7g; Additional file 1: Figure S6A), while the cell viability further decreased in the PDTC-treated cells or P65-knocked-down cells (Additional file 1: Figure S6B & C). Thus, these results demonstrated that IVM increased the sensitivity of the cells to VCR by inhibiting the activation of NF-κB.

To determine whether the downregulation of P-gp by IVM was directly mediated by NF-κB’s transcriptional activity, we co-transfected HCT-8 cells with the reporter vector for MDR1 promoter and the expression vector for P65. The downregulated MDR1 promoter activity by IVM was recovered when P65 was overexpressed, but was further inhibited when P65 was knocked down (Fig. 7h; Additional file 1: Figure S6D). Furthermore, we performed chromatin immunoprecipitation (ChIP) to determine whether P65 directly bound MDR1 promoter region. We observed that indeed P65 antibody could pull down the MDR promoter region, and IVM treatment reduced the binding of P65 with the MDR1 promoter region in HCT-8 cells (Fig. 7i). Thus, the inhibition of P-gp expression by IVM was mediated by the direct regulation of P-gp transcription by NF-κB. Consistently, we found that in the ADR-sensitive/resistant K562 cells, IVM also inhibited the activation of EGFR/ERK/Akt/NF-κB pathway and inhibited the expression of P-gp (Additional file 1: Figure S7A). Altogether, these findings demonstrated that the increase of sensitivity to VCR by IVM in the cells resulted from the downregulation of P-gp by the inhibition of EGFR/ERK/Akt/NF-κB pathway.

Consistently, in the solid tumor model, the levels of p-EGFR, p-ERK, p-Akt, p-P65, and P-gp were significantly downregulated in the mice treated with IVM or IVM plus VCR (Additional file 1: Figure S7B & C), indicating that IVM efficiently inhibited the EGFR/ERK/Akt/NF-κB signaling pathway *in vivo*.

### Ivermectin directly binds to EGFR

**Fig. 8:**
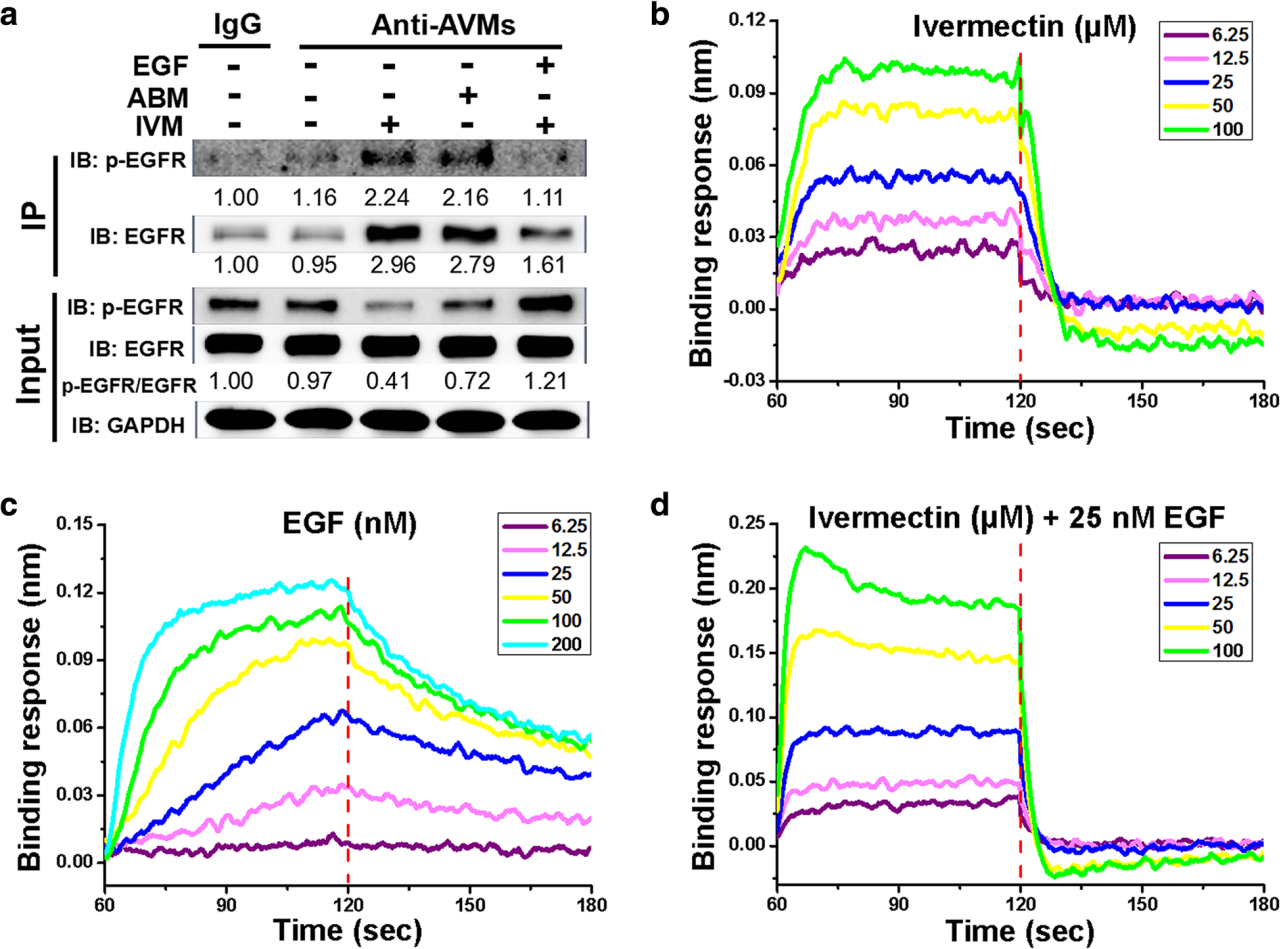
Ivermectin directly binds to EGFR. **a** Co-immunoprecipitation assay in HCT-8 cells treated with 3 μM IVM for 4 h with or without 10 nM EGF pretreatment for 2 h. Cell lysates were immunoprecipitated with non-specific IgG or anti-AVMs antibody that can cross-react with ABM and IVM. HCT-8 cells treated with ABM serve as positive control for the IP with anti-AVMs antibody. ‘IgG’ indicates the vehicle-treated cell lysates immunoprecipitated with non-specific IgG. ‘Input’ indicates the whole cell lysates. **b-d** Binding response (nm) between EGFR extracellular domain and different concentrations of ivermectin (IVM) (**b**), epidermal growth factor (EGF) (**c**) or the mixture of different concentrations of IVM with 25 nM EGF (**d**) was measured by Octet RED96 system. Abbreviations: IVM, ivermectin; ABM, abamectin; AVMs, avermectins; EGF, epidermal growth factor; EGFR, epithelial growth factor receptor. Western blots are representative of two independent experiments

Then, we sought to determine whether IVM inhibited EGFR by directly interacting with EGFR in HCT-8 cells to inhibit it. We found that EGFR and p-EGFR were co-immunoprecipitated with IVM using the antibody recognizing AVMs (anti-AVMs), which can react with both abamectin (ABM) and ivermectin (IVM), and the precipitated EGFR and p-EGFR levels were reduced by EGF pretreatment (Fig. 8a). Furthermore, binding affinity between IVM and EGFR extracellular domain was measured in an Octet RED96 system, which was based on the biolayer interferometry technology. The result of the real time analysis showed that there was a specific interaction between IVM and EGFR [Kd = 28 μM, coefficient of determination (r^2^) = 0.99] (Fig. 8b). EGF was used as a positive control with Kd = 19 nM [coefficient of determination (r^2^) = 1] (Fig. 8c). However, the interaction between IVM and EGFR was inhibited when EGF was present [Kd = 67 μM > 28 μM, coefficient of determination (r^2^) = 1] (Fig. 8d). Thus, IVM could directly interact with EGFR extracellular domain, albeit with a lower binding affinity compared with its endogenous ligand EGF, and IVM may compete with EGF for the same binding site. Altogether, IVM could directly bind with EGFR and the binding site was probably the same with EGF.

## Discussion

Ivermectin (IVM) and some other avermectins had well-known anti-parasitic activity. In this study, we showed that IVM had no significant toxic effect on the tumor cells at relative low concentrations. With IVM treatment, the sensitivity of the resistant tumor cells, including not only the solid tumor cells, such as HCT-8 and MCF-7 cells, but also the leukemia cells, such as K562 cells, to the chemotherapeutic drugs was recovered to almost the same level as that of the sensitive cells both *in vitro* and *in vivo*.

Some of the AVMs including IVM were found to have the anti-cancer effects [36–38]. In the literature, IVM was used only against the drug-sensitive tumor cells in xenograft animal models [20, 39, 40]. In these studies, higher doses of IVM (up to 10 mg/kg body weight, i.p.) could directly inhibit the tumor growth. However, in our study, it is the first time to show that IVM could reverse multidrug resistance of cancer cells *in vivo*. Moreover, the dose (2 mg/kg body weight, i.p.) we used in the mice was lower than those used to directly inhibit tumor growth. Furthermore, the dose of 2 mg/kg was shown to be approximately corresponding to what is given as anthelmintic agent in humans [17, 41]. Thus, it might be practical to use IVM in the clinic to overcome the resistance of tumor cells to chemotherapeutic drugs.

It was reported that IVM induced the expression of P-gp in the mouse hepatocytes and intestinal cells [42, 43]. However, there is no report on whether IVM affected P-gp expression when it was used to treat cancers. In our study, we revealed that IVM inhibited the P-gp expression in all tested cancer cells and the xenograft models, and the reversal of MDR by IVM was largely due to the downregulation of P-gp expression as shown by the results of overexpression of P-gp in the cells (Fig. 4). The different effects of IVM on P-gp expression in mouse hepatocytes, intestinal cells, and human cancer cells may be due to the difference of tissues, animal species, and the drug doses used. Also, there were several reports showing that the anti-parasitic drug IVM was the P-gp substrate, and P-gp played a role in the IVM resistance in parasites and altered function of P-gp in blood-brain barrier would result in severe IVM-induced neurotoxicity [44–46]. In our study, we indeed found that IVM inhibited P-gp function (Fig. 4c and g), but the main reversal mechanism of IVM was through EGFR signaling pathway (Figs. 5 and 6).

EGFR is an important factor that enhances the malignancy of drug-resistant breast cancer cells and mediates the resistance of the prostate cancer cells to chemotherapeutic drugs [33, 34]. The current study revealed, for the first time, that IVM directly interacted with human EGFR extracellular domain to inhibit EGFR. A previous study showed that EGFR inhibitor could reverse MDR by downregulating P-gp expression [47]. However, it was not clear how EGFR regulated P-gp expression in cancer cells. In our study, by using the activators and inhibitors of EGFR/ERK/Akt/NF-κB signaling pathway as well as overexpression or knockdown of key signaling molecules, we demonstrated that IVM downregulated the expression of P-gp at least largely through inhibiting the activation of the EGFR/ERK/Akt/NF-κB pathway in tumor cells. In addition, we showed that NF-κB directly regulated the expression of P-gp as a transcriptional factor.

It has been reported that IVM inhibited Wnt pathway [19]. However, we found that the Wnt/β-catenin did not play a role in the reversal effects of IVM or had effect on the P-gp expression (Additional file 1: Figure S8A-C). In addition, IVM has been shown to inhibit mTOR pathway [20]. However, we found that neither IVM nor VCR affected mTOR pathway (Additional file 1: Figure S8D). Furthermore, the activation of mTOR by the adenovirus or the inhibition of mTOR by rapamycin did not affect the reversal effect of IVM (Additional file 1: Figure S8E and F). Thus, the EGFR pathway is the major mechanism of the reversal effect of IVM on the resistant cancer cells.

In this study, we suggested that the effects of IVM were mainly mediated through inhibiting the EGFR pathway to reduce the transcription and expression of P-gp in the cancer cells. Thus, IVM may produce much stronger and longer reversal effect compared with those that only directly inhibit P-gp activity such as VRP. We observed that even after IVM was removed from the medium, its reversal effect still persisted (Fig. 6h). In addition, we showed that the reversal effect of an EGFR inhibitor LAP was mediated by the inhibition of EGFR to downregulate the expression of P-gp (Fig. 6f and g). This result is contrary to earlier reports showing that the reversal effect of LAP was mediated by its direct inhibition of P-gp activity [48, 49]. Thus, we propose that EGFR may serve as a new and effective target for developing novel reversal agents.

## Conclusions

In summary, our study reveals that IVM increased the sensitivity of tumor cells, including the drug-sensitive or resistant cancer cells, solid tumor cells or leukemia cells, to the chemotherapeutic drugs. It is the first time to show that IVM could reverse multidrug resistance *in vivo*. Mechanistically, IVM directly interacts with the extracellular domain of EGFR, and reverses the drug resistance by inhibiting the EGFR/ERK/Akt/NF-κB pathway to downregulate the expression of P-gp. Therefore, we propose that IVM might be used clinically as a therapy to resolve the MDR problem, given that IVM has already been approved in human use.

## Additional files

Additional file 1: **Figure S1.** Ivermectin increased the sensitivity of the cells to mitomycin C and adriamycin. **Figure S2.** The anticancer effects of ivermectin in a leukemia mice model. **Figure S3.** Ivermectin increases the sensitivity of the cells to vincristine by inhibiting P-gp expression. **Figure S4.** Ivermectin decreased P-gp expression by inhibiting the ERK/Akt activation. **Figure S5.** Ivermectin inhibits P-gp expression through EGFR/ERK/Akt/NF-κB pathway. **Figure S6.** IVM increased the sensitivity of HCT-8 cells to VCR by inhibiting the activation of NF-κB. **Figure S7**. Ivermectin inhibited the activation of EGFR/ERK/Akt/NF-κB and P-gp expression in K562 cells and HCT-8 xenografts in nude mice. **Figure S8**. The reversal effects of ivermectin had no related to Wnt/β-catenin and mTOR pathway. (PDF 1407 kb)

## Data Availability

The datasets used and analyzed during the current study are available from the corresponding author on reasonable request. All data supporting the conclusions of this article are included within the article and additional files.

## Abbreviations

ABC: ATP-binding cassette
ABM: Abamectin
ADR: Adriamycin
AVM: Avermectins
CML: Chronic myeloid leukemia
Co-IP: Co-immunoprecipitation
EGFR: Epidermal growth factor receptor
IVM: Ivermectin
MC: Mitomycin C
MDR: Multiple drug resistance
P-gp: P-glycoprotein
SSA: Super Streptavidin
VCR: Vincristine
WB: Western blotting
