# Supplementary material for: Ivermectin reverses the drug resistance in cancer cells through EGFR/ERK/Akt/NF-κB pathway
Source: J Exp Clin Cancer Res. 2019 Jun 18;38:265. doi: 10.1186/s13046-019-1251-7 (PMC6580523; doi:10.1186/s13046-019-1251-7)
Supplement: Supplementary file 1 — Figure S1. Ivermectin increased the sensitivity of the cells to mitomycin C and adriamycin. Figure S2. The anticancer effects of ivermectin in a leukemia mice model. Figure S3. Ivermectin increases the sensitivity of the cells to vincristine by inhibiting P-gp expression. Figure S4. Ivermectin decreased P-gp expression by inhibiting the ERK/Akt activation. Figure S5. Ivermectin inhibits P-gp expression through EGFR/ERK/Akt/NF-κB pathway. Figure S6. IVM increased the sensitivity of HCT-8 cells to VCR by inhibiting the activation of NF-κB. Figure S7. Ivermectin inhibited the activation of EGFR/ERK/Akt/NF-κB and P-gp expression in K562 cells and HCT-8 xenografts in nude mice. Figure S8. The reversal effects of ivermectin had no related to Wnt/β-catenin and mTOR pathway. (PDF 1407 kb) [file 13046_2019_1251_MOESM1_ESM.pdf]

Supplementary figures

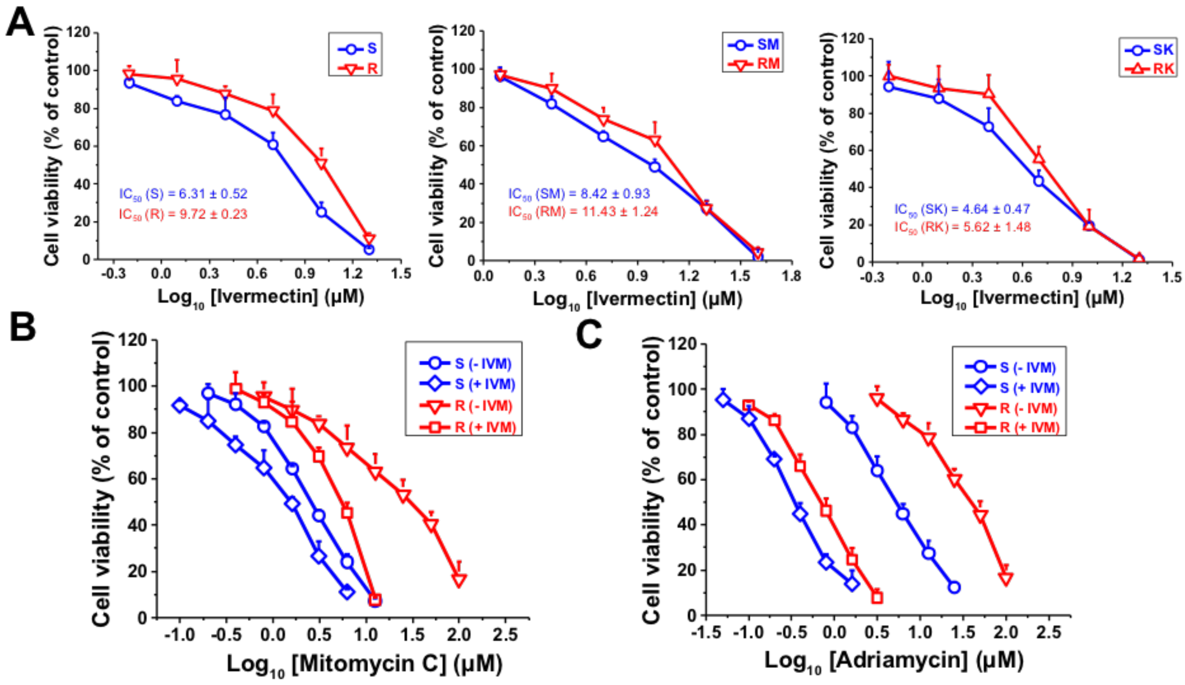

Figure S1. Ivermectin increased the sensitivity of the cells to mitomycin C and adriamycin. **A**, The cell viability of sensitive or resistant human colorectal cancer HCT-8 cells, breast cancer MCF-7 cells and chronic myelogenous leukemia K562 cells treated with different concentrations of IVM for 48 h was determined by MTT assay. **B** and **C**, The viability of the HCT-8 cells exposed to different concentrations of mitomycin C (**B**) or adriamycin (**C**) with or without 3 μM ivermectin (IVM). Cells treated with vehicle serve as a blank control. Abbreviations: IVM, ivermectin. S, vincristine-sensitive HCT-8 cells; R, vincristine-resistant HCT-8 cells; SM, adriamycin-sensitive MCF-7 cells; RM, adriamycin-resistant MCF-7 cells; SK, adriamycin-sensitive K562 cells; RK, adriamycin-resistant K562 cells. All experiments were conducted in quintuplicates and data were expressed as the mean ± SD (n = 5).

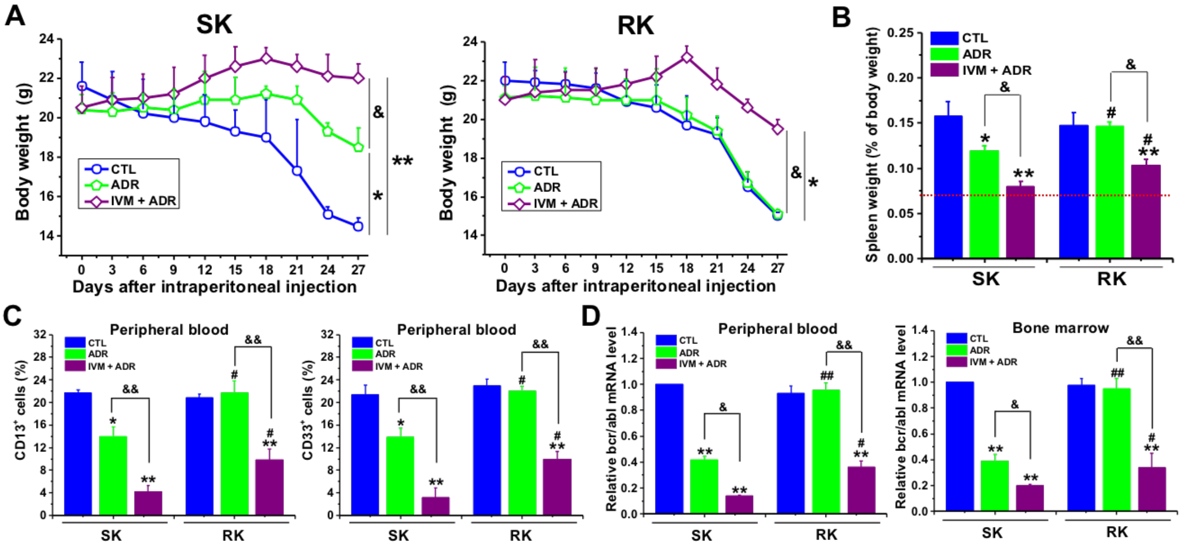

Figure S2. The anticancer effects of ivermectin in a leukemia mice model. The NOD/SICD mice were injected through tail vein with 2×10^7 K562 cells, which are sensitive or resistant to adriamycin (ADR). Then, the mice were treated with 0.3 mg/kg ADR alone or combined with 2 mg/kg ivermectin (IVM) by intraperitoneal injection daily for 27 days. **A** and **B**, Body weight (**A**) and spleen weight **(B)** of the mice were determined. The red dash line presents the normal level of the spleen weight of the mice. **C**, The percentage of cells stained positive with cell surface marker CD13 or CD 33 in peripheral blood was determined by using flow cytometry. **D**, mRNA levels of bcr/abl in peripheral blood and bone marrow were determined by using qPCR. Mice treated with vehicle serve as control. Abbreviations: BW, body weight; CTL, control; IVM, ivermectin; ADR, adriamycin; SK, adriamycin-sensitive K562 xenograft; RK, adriamycin-resistant K562 xenograft. Data were expressed as the mean ± SD (n = 6 mice each group). Statistical significances were determined using one-way ANOVA followed by Dunnett's test. $^{*}P < 0.05$, $^{**}P < 0.01$, compared with the respective vehicle controls (blue columns/lines); $^{\#}P < 0.05$, $^{\#\#}P < 0.01$ compared with the corresponding columns with the same color in the SK group; $^{\&}P < 0.05$, $^{\&\&}P < 0.01$, comparison between the two columns or lines.

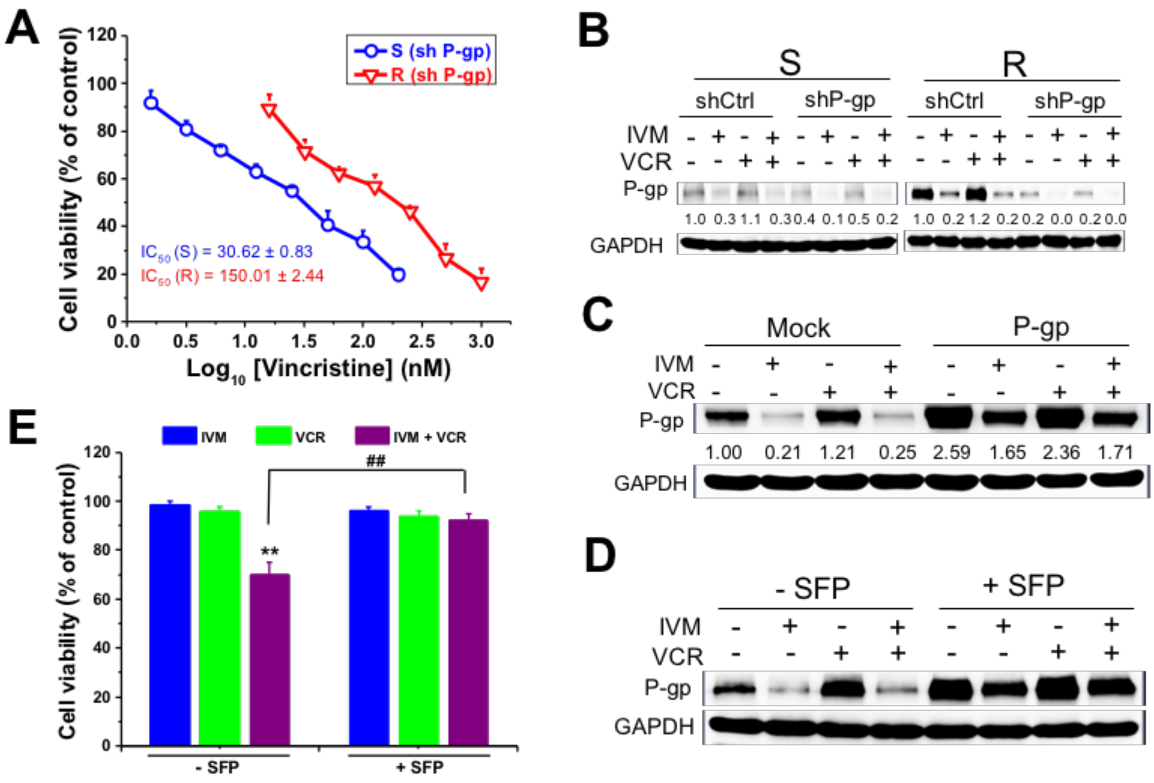

Figure S3. Ivermectin increases the sensitivity of the cells to vincristine by inhibiting P-gp expression. The cell viability (**A** and **E**) and P-gp expression level (**B-D**) of the VCR-sensitive/resistant HCT-8 cells (**A** and **B**) or VCR-resistant cells (**C-E**) transfected with the plasmid pGenesil-P-gp (P-gp shRNA) (**A** and **B**), the plasmid pcDNA3.1 (+)-P-gp (**C**) and treated with different concentrations of VCR (**A**) or 25 nM VCR and/or 3 μM IVM (**B-E**) in the presence or absence of 2.5 μM sulforaphane (SFP) (**D** and **E**) for 48 h were determined**.** SFP is an activator of the transcription factor Nrf2. Cell viability was determined by MTT method and protein expression was detected by Western blotting analysis using GAPDH as a loading control. Cells transfected with control shRNA (shCtrl)/empty vector pcDNA3.1(+) (mock) or treated with vehicle serve as control. Abbreviations: IVM, ivermectin; SFP, sulforaphane; VCR, vincristine. Western blots are the representative of two independent experiments. Data in **A** and **E** were expressed as mean ± SD (n = 5). Statistical significances in **E** were determined using one-way ANOVA followed by Dunnett's test. $^{**}P < 0.01$, compared with the respective vehicle controls; $^{\#\#}P < 0.01$, comparison between the indicated two columns.

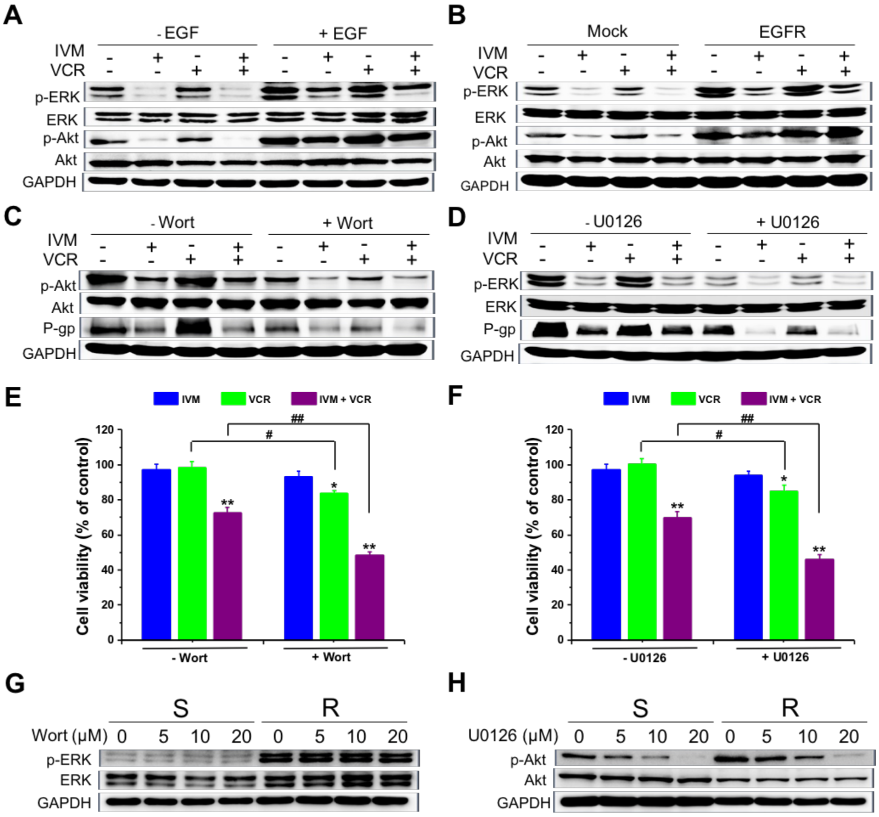

Figure S4. Ivermectin decreased P-gp expression by inhibiting the ERK/Akt activation. A-F, The expression levels of the proteins (**A-D**) and cell viability (**E** and **F**) in the VCR-resistant HCT-8 cells untransfected (**A, C-F**) or transfected with pcDNA3.1(+)-EGFR (**B**), treated with 25 nM VCR and/or 3 μM IVM and with or without 8 nM EGF (**A**), 10 μM Wort, an Akt inhibitor (**C** and **E**), or 10 μM U0126, an ERK inhibitor (**D** and **F**) for 48 h was determined by Western blotting analysis. **G** and **H,** The expression levels of p-ERK/ERK and p-Akt/AKT of the HCT-8 cells treated with Wort (**G**) or U0126 (**H**) were detected. Cell viability was detected by MTT assay and the protein expression levels were detected by Western blotting analysis using GAPDH as internal control. Cells treated with vehicle serve as control. Abbreviations: EGF, epidermal growth factor; IVM, ivermectin; VCR, vincristine; Wort, wortmannin; S, vincristine-sensitive HCT-8 cells; R, vincristine-resistant HCT-8 cells. Data in **A-D** and **G-H** are the representative of two independent experiments. Data in **E** and **F** represent the percentage of respective control values (mean ± SD, n = 5). Statistical significances were determined using one-way ANOVA followed by Dunnett's test. $^{*}P < 0.05$, $^{**}P < 0.01$, compared with the respective controls; $^{\#}P < 0.05$, $^{\#\#}P < 0.01$, comparison between the two columns.

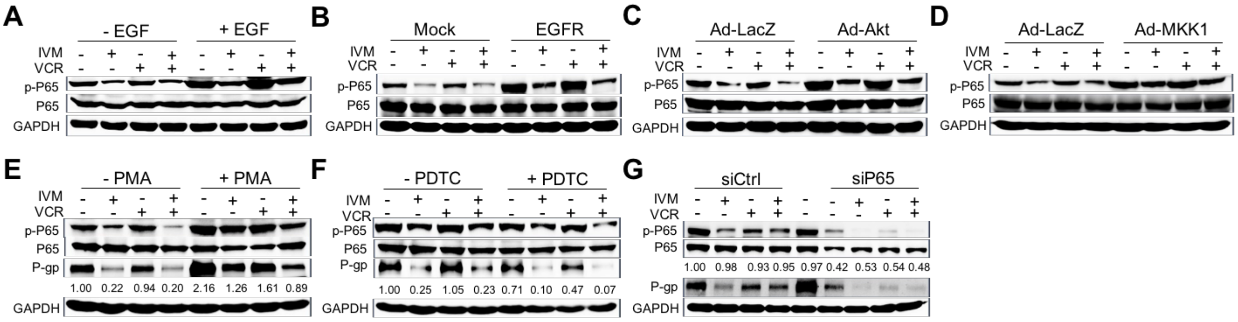

Figure S5. Ivermectin inhibits P-gp expression through EGFR/ERK/Akt/NF-κB pathway. The VCR-resistant HCT-8 cells transfected with the plasmid (**B** and **G**), or infected by the recombinant adenovirus (**C** and **D**), or neither transfected nor infected **(A, E, and F)** were used to do the tests. **A-G**, The expression levels of the proteins in the untransfected cells treated with 25 nM VCR and/or 3 μM IVM in the presence or absence of 8 nM EGF (**A**), 10 nM PMA, an NF-κB activator (**E**) or 10 μM PDTC, an NF-κB inhibitor (**F**), or the cells transfected with pcDNA3.1(+)-EGFR (**B**) or P65 siRNA (**G**) or the cells infected by recombinant adenoviral vector expressing Ad-Akt (**C**) or Ad-MKK1 (**D**) treated with 25 nM VCR and/or 3 μM IVM for 48 h were detected by Western blotting analysis using GAPDH as internal control. Cells treated with vehicle, transfected with empty vector pcDNA3.1(+) (mock)/control siRNA (siCtrl) or infected with Ad-LacZ serve as control. Abbrevaitons: IVM, ivermectin; VCR, vincristine; EGF, epidermal growth factor; PDTC, pyrrolidinedithiocarbamic acid; PMA, phorbol-12-myristate-13-acetate. Data are the representative of two independent experiments.

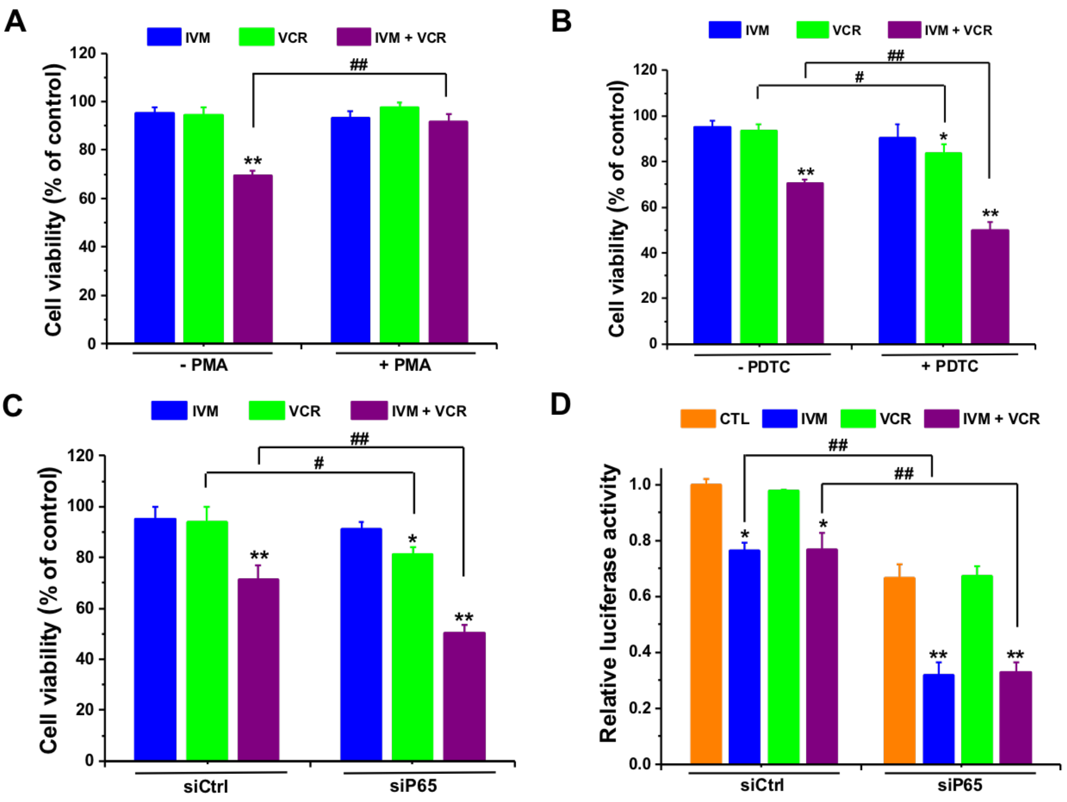

Figure S6. IVM increased the sensitivity of HCT-8 cells to VCR by inhibiting the activation of NF-κB. A-D, The cell viability (**A-C**) and the relative MDR1 promoter activity (**D**) of the VCR-resistant HCT-8 cells untransfected (**A** and **B**) or transfected with the P65 siRNA (**C** and **D**) treated with 3 μM IVM and/or 25 nM VCR in the presence or absence of 10 nM PMA, an NF-κB activator (**A**) or 10 μM PDTC, an NF-κB inhibitor (**B**) for 48 h. Cell viability was detected by MTT assay. Relative MDR1 promoter activity was determined by *Gaussia* luciferase activity normalized to the transfection control, i.e., secreted alkaline phosphatase (SeAP). Cells treated with vehicle, or transfected with control siRNA (siCtrl) serve as control. Abbrevaitons: IVM, ivermectin; VCR, vincristine; PDTC, pyrrolidinedithiocarbamic acid; PMA, phorbol-12-myristate-13-acetate. Data in **A-C** represent the percentage of respective control values (mean ± SD, n = 5). Data in **D** are expressed as fold change of the activity over the control from the corresponding siCtrl group (mean ± SD, n = 3). Statistical significances in **A-D** were determined using one-way ANOVA followed by Dunnett's test. $^{*}P < 0.05$, $^{**}P < 0.01$, compared with the respective vehicle controls; $^{\#}P < 0.05$, $^{\#\#}P < 0.01$, comparison between the two columns.

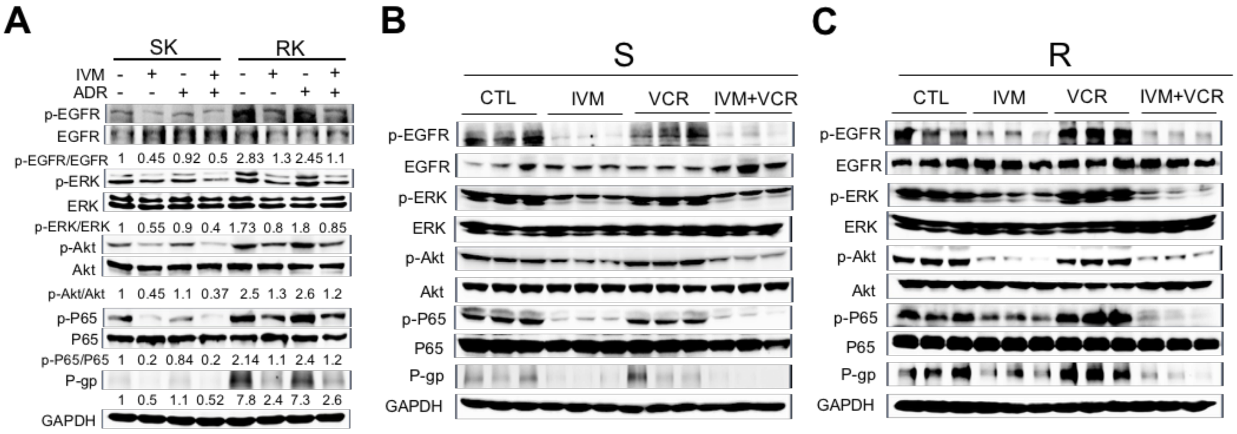

Figure S7. **Ivermectin inhibited the activation of EGFR/ERK/Akt/NF-κB and P-gp expression in K562 cells and HCT-8 xenografts in nude mice**. **A**, The expression levels of the proteins in K562 cells after treated with 250 nM adriamycin (ADR) and/or 2.5 μM ivermectin (IVM) for 48 h. **B** and **C,** The expression levels of proteins in HCT-8 xenografts in nude mice. The nude mice were injected subcutaneously with 1×10^7 VCR-sensitive HCT-8 cells (**B**) or VCR-resistant HCT-8 cells (**C**). When the tumor reached to about 100 mm^3, the mice were treated with IVM (2 mg/kg) and/or VCR (0.2 mg/kg) by intraperitoneal injection daily for 27 days. The protein expression levels were detected by Western blotting analysis using GAPDH as a loading control. Cells treated with vehicle serve as control. Abbreviations: IVM, ivermectin; VCR, vincristine; ADR, adriamycin; SK, adriamycin-sensitive K562 cells; RK, adriamycin-resistant K562 cells; S, vincristine-sensitive HCT-8 xenograft; R, vincristine-resistant HCT-8 xenograft. Western blots are the representative of two independent experiments.

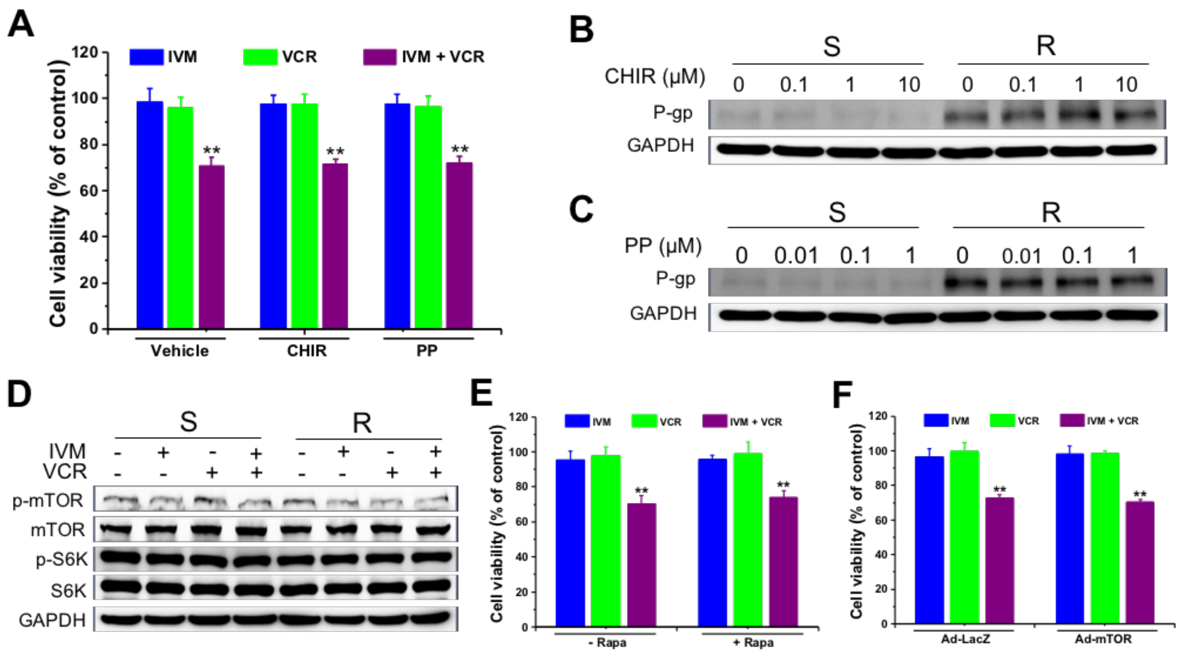

Figure S8. **The reversal effects of ivermectin had no related to Wnt/β-catenin and mTOR pathway.** The VCR-sensitive/resistant HCT-8 cells uninfected (**A-E**) or infected by recombinant adenovirus (**F**) were used to do the tests. **A**, The cell viability of the resistant cells treated with 25 nM vincristine (VCR) and/or 3 μM ivermectin (IVM) in the presence of 10 μM CHIR-99021 (CHIR), an activator of Wnt/β-catenin pathway or 1 μM pyrvinium pamoate (PP), an inhibitor of Wnt/β-catenin pathway, for 48 h were determined. **B-D,** The expression levels of the proteins in the cells treated with CHIR (**B**) or PP (**C**), or 25 nM VCR and/or 3 μM IVM (**D**) were detected. **E** and **F**, The viability of the resistant cells treated with or without 10 μM rapamycin (Rapa), an mTOR inhibitor (**E**) or the cells infected by recombinant adenovirus expressing HA-tagged mTOR (Ad-mTOR-WT) (**F**), treated with 25 nM VCR and/or 3 μM IVM for 48 h were determined. Cell viability was detected by MTT assay and the protein expression levels were detected by Western blotting analysis using GAPDH as internal control. Cells treated with vehicle or recombinant adenovirus expressing Ad-LacZ serve as control. Abbreviations: IVM, ivermectin; VCR, vincristine; S, vincristine-sensitive HCT-8 cells; R, vincristine-resistant HCT-8 cells; CHIR, CHIR 99021; PP, pyrvinium pamoate; Rapa, rapamycin. Western blots in **B-D** are representative of two independent experiments. Data in **A**, **E,** and **F** represent the percentage of respective control values (mean ± SD, n = 5). Statistical significances in **A**, **E,** and **F** were determined using one-way ANOVA followed by Dunnett's test. $^{**}P < 0.01$, compared with the respective controls.
